# Residual Interpolation Integrated Pixel-by-Pixel Adaptive Iterative Process for Division of Focal Plane Polarimeters

**DOI:** 10.3390/s22041529

**Published:** 2022-02-16

**Authors:** Jie Yang, Weiqi Jin, Su Qiu, Fuduo Xue, Meishu Wang

**Affiliations:** MOE Key Laboratory of Optoelectronic Imaging Technology and System, Beijing Institute of Technology, Beijing 100081, China; 3120170328@bit.edu.cn (J.Y.); edmondqiu@bit.edu.cn (S.Q.); 3120195334@bit.edu.cn (F.X.); 3120200573@bit.edu.cn (M.W.)

**Keywords:** demosaicing, division of focal plane polarimeters, iteration, residual interpolation

## Abstract

Residual interpolations are effective methods to reduce the instantaneous field-of-view error of division of focal plane (DoFP) polarimeters. However, their guide-image selection strategies are improper, and do not consider the DoFP polarimeters’ spatial sampling modes. Thus, we propose a residual interpolation method with a new guide-image selection strategy based on the spatial layout of the pixeled polarizer array to improve the sampling rate of the guide image. The interpolation performance is also improved by the proposed pixel-by-pixel, adaptive iterative process and the weighted average fusion of the results of the minimized residual and minimized Laplacian energy guide filters. Visual and objective evaluations demonstrate the proposed method’s superiority to the existing state-of-the-art methods. The proposed method proves that considering the spatial layout of the pixeled polarizer array on the physical level is vital to improving the performance of interpolation methods for DoFP polarimeters.

## 1. Introduction

Polarization, amplitude, wavelength, and phase are the four most important physical characteristics of light. Polarimeters can obtain the intensity (amplitude) and polarization information of the target scene to calculate polarization parameters such as the Stokes vector, the degree of linear polarization (DoLP), and the angle of polarization (AoP). Subsequently, the target contrast enhancement, stealth target detection, and deduction of characteristic information such as surface shape, roughness, and spatial attitude can be achieved. Polarization imaging technology is, therefore, extensively used in target detection and classification [1,2,3], three-dimensional shape reconstruction [4,5,6], remote sensing observation [7,8,9], and medical biological imaging [10,11].

**Figure 1:**
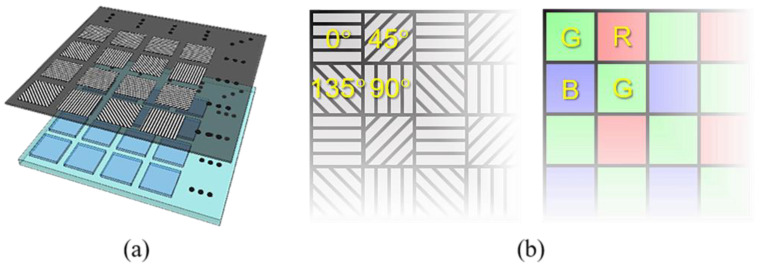
Schematic diagrams of DoFP polarimeter and the color filter array. (**a**) Illustrates the structure of DoFP polarimeters; (**b**) presents the spatial layout of the pixeled polarizer array and the color filter array.

The increasingly mature nanomanufacturing technology and the urgent need to simultaneously detect polarization information promote the rapid development of miniaturized and compact division of focal plane (DoFP) polarimeters [12,13,14,15]. Companies such as FLIR [16], 4D Technology [17], and LUCID Vision Labs [18] have successively launched DoFP polarimeter products that can be used for precision measurement. This polarimeter integrates a CCD/CMOS sensor and an aluminum nanowire polarizer filter array with a similar pixel structure, as in the imaging focal plane array (FPA) (Figure 1a). The output of this polarimeter is an incompletely sampled mosaic image with four polarization channels of 0°, 45°, 90°, and 135°. Each channel corresponds to a different instantaneous field of view (IFOV) due to the spatial dislocation arrangement. When the polarization information of these four channels is directly used to calculate the polarization parameters, the spatial resolution of the calculated polarization parameter image is reduced to 1/4 of that of FPA. Further, errors (such as the sawtooth effect) will be present in regions with abundant edge and texture. These phenomena form so-called the inherent IFOV error of DoFP polarimeters.

Reducing the IFOV error and restoring the spatial resolution is generally achieved by interpolating the output mosaic image of cameras using demosaicing methods. DoFP polarimeters shares the same principle of division of focal plane as the Bayer color camera. Therefore, the IFOV error formation mechanism of these two cameras is the same. Research into demosaicing methods for DoFP polarimeters usually refers to the earlier color image demosaicing methods [19,20,21,22,23,24,25,26,27]. In recent years, several color image demosaicing methods have been effectively transferred to DoFP polarimeters. These methods can be classified as:

(1) Methods of independently interpolating using single-channel, which mainly include polynomial interpolation methods (bilinear [28,29,30,31], bicubic, bicubic spline [32,33], etc.) and edge directionality interpolation methods (gradient [34,35], smoothness [36], etc.). they are easy to implement, but their performance is mediocre.
(2) Methods of interpolating using other channels as reference images, which mainly include correlation-based interpolation methods [37,38,39,40] and residual interpolation methods [41,42,43,44,45]. They are balanced in performance and stability and are the main topic of this paper. Recently, some heuristic algorithms (e.g., heuristic validation mechanisms) have been shown to find some important regions in traditional images [46], and they are expected to be combined with the residual interpolation algorithms to further improve interpolation performance.
(3) Learning-based methods, which mainly include optimization-based methods [47], sparse representation-based methods [48], and deep learning-based methods [49,50,51]. They are considered to have the best performance on the published datasets, but their algorithm designs do not directly correspond to the DoFP polarimeter model, and the current open-access datasets contain very limited polarization scenarios.

The residual interpolation demosaicing methods can utilize the similar edge and texture features of the four channel images. Two channels are selected as the input image and the guide image to generate the initial estimate using the guide filter, and interpolation is executed in the residual domain containing less high-frequency information (where the residual is the difference between the observed image and the tentatively estimated image). This method has been proven to have a better demosaicing performance than other traditional polarization interpolation methods [41,42,43,44,45].

Two residual interpolation methods to demosaic DoFP polarimeters, with minimized residual (PRI) [41,42] and minimized Laplacian energy (MLPRI) [43], have been reported. However, these two methods did not thoroughly consider the inherent differences in the spatial sampling modes of DoFP polarimeters and Bayer color cameras. The following problems are present in the selection and preprocessing of the guide image:

(1) The spatial layout of four channels in the pixeled polarizer array is not thoroughly considered when selecting the polarization direction of the guide image in PRI and MLPRI. In the color filter array, the sampling rate of G channel is 50%, which is twice that of the R and B channels (Figure 1b). Therefore, the G channel is usually interpolated first, and its interpolation result is also used as a reference image when interpolating the R and B channels, which makes the performance of residual interpolation methods better than the performance of traditional single-channel interpolation methods. In contrast, the sampling rates of the four channels in the pixeled polarizer array of DoFP polarimeters are equal, so there is no specific dominant direction. The existing PRI or MLPRI intuitively selects the same channel as the input image or the 0° channel as the guide image. The selected guide image does not have an advantage in terms of sampling rate, which makes the improvements in the performance of PRI and MLPRI insignificant compared with the single-channel interpolation methods.
(2) The guide filter requires the guide image to have the same high resolution as the interpolation result. High-resolution images cannot be directly obtained during the actual polarization imaging process. Therefore, the guide image is usually generated by preprocessing the low-resolution observed image. Referring to color image demosaicing, PRI and MLPRI use basic interpolation methods, such as bilinear and bicubic interpolation, to up-sample the observed image and generate the guide image. However, when the sampling rate of the observed image is low, the guide image generated by this preprocessing may exhibit large errors in regions with an abundant edge and texture. This error will be transmitted to the tentatively estimated image, and further affect the quality of the output interpolation result.

Looking at this problem, this study proposed a residual interpolation method integrating a pixel-by-pixel adaptive iterative process for DoFP polarimeters (PAIPRI). Compared with the previously published PRI and MLPRI, innovative designs of the proposed method have been carried out, focusing on the following aspects:

(1) We proposed a new guide-image selection strategy. We considered the spatial layout of the pixeled polarizer array, and chose different channels as the guide image for the pixels in different spatial positions. In addition, cooperating with the different sizes and directions of the filter window, the sampling rate of the adopted guide image in the filter window increased to 50%.
(2) We designed a pixel-by-pixel adaptive iterative process based on residual interpolation. The guide image and the interpolation result were adaptively updated pixel-by-pixel through two interrelated iterative processes to improve the demosaicing performance of the output interpolation result.
(3) We performed an adaptively weighted average fusion on the local iterative optimal results of the two guide filters, and minimized residual and minimized Laplacian energy, to make the interpolation results better.
(4) Unlike the current mainstream learning-based methods, our algorithm is completely physical-fact-based and can explain the down-sampling process of the DoFP polarimeter. Furthermore, the focus on the improving imaging system makes our algorithm completely independent of the polarized images being processed, making it more robust to unseen scenes.

We conducted comparison experiments using both the open-access dataset images collected by a division-of-time polarimeter and the indoor and outdoor scene images collected by a real-world DoFP polarimeter to analyze and compare the demosaicing performance of the proposed method and the six previously published methods in both visual comparison and objective evaluation.

The remainder of this paper is organized as follows: Section 2 summarizes the previously published demosaicing methods for DoFP polarimeters and the basic polarization theory. Section 3 describes the selection strategy and preprocessing of the guide image. Section 4 presents the principle and process of the proposed PAIPRI method in detail. Section 5 reports the visual comparison and objective evaluation of the proposed PAIPRI method using both the open-access dataset images collected by a division-of-time (DoT) polarimeter and the indoor and outdoor scene images collected by a real-world DoFP polarimeter. Finally, Section 6 concludes the study.

## 2. Related Works

### 2.1. Demosaicing Methods for DoFP Polarimeters

Since Ratliff et al. [28] first discussed the method of reducing the IFOV error for DoFP polarimeters in 2006, more than ten demosaicing methods have been reported for DoFP polarimeters. These methods can be classified as methods of interpolating independently using single-channel methods of interpolating, using other channels as reference images and learning-based methods.

#### 2.1.1. Methods of Interpolating Independently Using Single-Channel

Methods of interpolating independently using single-channel performs analysis and interpolation independently on each channel image, which mainly include polynomial interpolation methods and edge directionality interpolation methods.

The polynomial interpolation method is based on a spatially invariant non-adaptive linear filter. This method estimates the polarization information of un-sampled pixels using the sampling polarization information in the neighborhood by polynomial fitting. This method was first studied due to its low computational complexity. Ref. [30] used the sampling pixels of the same polarization direction in a 3 × 3 neighborhood to estimate the unsampled polarization information of the center pixels through bilinear interpolation. Ref. [33] used the sampling pixels of the same polarization direction in a 5 × 5 neighborhood to estimate the three unsampled polarization information of the center pixels through weighted bilinear, bicubic, and bicubic spline interpolation. Moreover, Ref. [33] designed an approximated bicubic spline method to reduce computational complexity. Its low computational complexity and good reconstruction performance on low-frequency information make the polynomial interpolation method easy to implement on hardware platforms. Ref. [31] implemented the real-time bilinear interpolation of 1600 × 1200 images on FPGA at a speed of 50 frames/s. The adopted window sizes were highly correlated with the PSF function of the imaging system. The polynomial interpolation method performs well with low-frequency information. Increasing the polynomial order can inspire more accurate interpolation results. However, the polynomial interpolation method is usually integrated into other demosaicing methods as a basic method because of its poor performance with high-frequency information.

The edge directionality interpolation method aims to perform polynomial interpolation along the edge instead of across the edge. The foremost task of this method is to accurately detect the edge direction in the incompletely sampled mosaic observed image. Ref. [35] used the horizontal, vertical, and diagonal gradients calculated in the 7 × 7 neighborhood as the criteria to detect the edge direction and performed bicubic convolution interpolation along the edge. Ref. [36] used the block variance, which characterizes the local smoothness, calculated in the 7 × 7 neighborhood, as the criterion to detect the edge direction, and performed bicubic interpolation along the edge. The window adopted sizes are highly correlated with the criterion calculation and the chosen interpolation method. The edge directionality interpolation method performs well in single large-scale edge. However, these criteria are extremely susceptible to IFOV errors, and are less able to discriminate complexly small-scale edges and textures.

#### 2.1.2. Methods of Interpolating Using Other Channels as Reference Images

The method of interpolating using other channels as reference images uses the linear relationship or the similar edge and texture features of the four polarization channels as the reference information source to perform interpolation, which mainly includes correlation-based interpolation methods and residual interpolation methods.

The correlation-based interpolation method uses the linear relationship between the four polarization channels as the reference information source to interpolate un-sampled pixels. Ref. [37] took at least one parameter of the intensity, the DoLP, or the AoP, as prior information to interpolate un-sampled pixels using the linear relationship between the four polarization channels as the reference information source. Ref. [39] designed an edge classifier based on the difference between the two channels and performed Newton polynomial interpolation along the recognized edge direction. Ref. [40] used the weighted fusion of the orthogonal and non-orthogonal polarization channels to interpolate. However, these methods need the’ targets prior information, or need the difference domain between the four polarization channels to be very smooth, and assumed the polarizers to be ideal. These assumptions lead to a simple linear correlation between the four analyzer channels. However, the pixeled polarizer array of DoFP polarimeters has an obvious spatially distributed non-ideality [52]. This non-ideality means that the demosaicing performance of these methods cannot be guaranteed for the real DoFP polarimeter images, and the application scenarios for these methods are extremely limited.

The residual interpolation method can utilize the similar edge and texture features among the four channel images. This method upsamples the input image using the guide filter by referring to the edge and texture features of the guide image, and executes interpolation in the residual domain with less high-frequency information. Ref. [41] selected the low-resolution observed image and high-resolution image of the same channel as the input image and the guide image, respectively, and generated the initial estimate through the minimized residual guide filter (RI). Ref. [43] selected the 0° channel image as the guide image and generated the initial estimate through the minimized Laplacian energy guide filter (MLRI). Ref. [44] selected the edge-aware intensity image generated by an edge detector using the intensity correlation as the guide image, and generated the initial estimate through MLRI. The biggest advantage of residual interpolation is that its parameter tuning is free from training images. This method can still obtain a generally better demosaicing performance, even in the new imaging scene. Moreover, this method has good interpretability for the spatial sampling modes of DoFP polarimeters. Nevertheless, the improper guide-image selection strategy in existing methods fails to fully utilize these advantages, and the performance of these methods can be further improved.

#### 2.1.3. Learning-Based Methods

Learning-based methods construct the sampling models [47] or demosaicing models [48,49,50,51] for DoFP polarimeters by training datasets. This can be achieved by convex optimization [47], dictionary learning [48], and convolutional neural networks (CNN) [49,50,51]. Although learning-based methods generally achieve a higher performance than traditional interpolation methods, they are highly data-dependent [44,53]. A large number of highly representative training images that cover a wide range of scenes are needed to ensure the generalization ability. However, it is very difficult to construct such a training dataset. Moreover, due to the spontaneous emission of infrared polarization devices, the images of DoT and DoFP infrared polarimeters are significantly different. The demosaicing performance of the network trained by DoT infrared images cannot be guaranteed for the real DoFP infrared polarimeters images [39].

### 2.2. Basic Theory of Polarization Imaging

The Stokes vector **S** [54] is typically used to describe the polarization characteristics of any light field and can be defined as:

(1) $$S={\left[\begin{array}{cccc} S_{0} & S_{1} & S_{2} & S_{3} \end{array}\right]}^{T},$$

where *S*_0_ is the total light intensity, *S*_1_ is the horizontal or vertical linear polarization component, *S*_2_ is the linear polarization component of +45° or −45° polarization directions, and *S*_3_ is the left- or right-handed circular polarization component. As the circular polarization component in natural scene radiation is extremely small, *S*_3_ is typically considered to be 0. Moreover, DoFP polarimeters only respond to linear Stokes parameters (i.e., *S*_0_, *S*_1_, and *S*_2_). Thus, *S*_3_ was omitted from the Stokes vector mentioned in this study.

DoLP and AoP are typically used to investigate the polarization states of the target scene. DoLP represents the proportion of the linearly polarized component to the total intensity of the light source, while AoP represents the angle between the polarization direction of the maximum incident light energy and the *x*-axis in the reference coordinate system. DoLP and AoP can be calculated using the Stokes vector as follows:

(2) $$\mathrm{DoLP}=\frac{\sqrt{S_{1}^{2}+S_{2}^{2}}}{S_{0}^{}},\;\mathrm{AoP}=\frac{1}{2}\arctan\left(\frac{S_{2}^{}}{S_{1}^{}}\right),$$

where DoLP ∊ [0, 1], and DoLP = 1 for linearly polarized light.

The process of polarization imaging and that of reconstructing the incident Stokes vector **S** using the output grayscale of the four polarization channels (that is, the measurement process) can be represented as [52]:

(3) $$\begin{array}{c} DN=\left(g\eta \right)M\cdot S,\\ S={\left(g\eta \right)}^{-1}M_{}^{†}\cdot DN, \end{array}$$

where **DN** is the output grayscale vector; *g* is the total gain of the sensor; *η* is the quantum efficiency of the sensor; **M** is the coefficient matrix, which characterizes the modulation effect of the pixelated polarizer on the incident Stokes vector, and $M^{†}$ is the pseudo-inverse matrix of **M**, $M^{†}={\left(M^{T}\cdot M\right)}^{-1}M^{T}$.

When the pixelated polarizer array of DoFP polarimeters satisfies the assumption of ideal polarizers (that is, the extinction ratios *ε*^2^ of the four pixels in each super-pixel approaches +∞, and polarization direction *θ* is equal to 0°, 45°, 90°, and 135°, respectively), the ideal normalized coefficient matrix **M***_ideal_* of a single super-pixel can be represented as follows:

(4) $$M_{ideal}=\frac{\tau }{2}\left[\begin{array}{ccc} 1 & 1 & 0\\ 1 & 0 & 1\\ 1 & -1 & 0\\ 1 & 0 & -1 \end{array}\right],$$

where *τ* is the transmittance coefficient of the pixelated polarizer.

## 3. Discussion of the Guide Image

### 3.1. Framework of the Residual Interpolation Methods for DoFP Polarimeters Demosaicing

**Figure 2:**
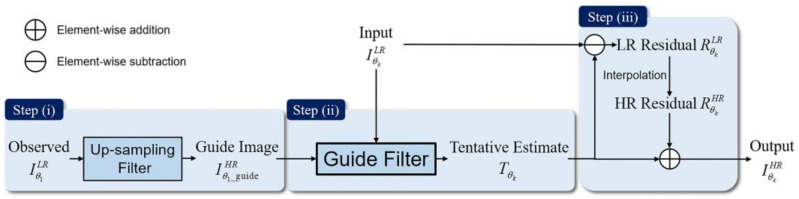
The framework of the residual interpolation methods for DoFP polarimeters demosaicing. Guided Filter represents RI or MLRI.

The residual interpolation method for DoFP polarimeters’ demosaicing assumes that the residual domain contains less high-frequency information, and executes interpolation in the residual domain using the simple polynomial interpolation method to generate a good demosaicing performance [41]. The previously published residual interpolation methods usually consist of three steps (Figure 2):

i Generate the guide image: We use an up-sampling filter to interpolate the low-resolution observed image $I_{\theta_{1}}^{LR}$ in a certain polarization direction to generate the guide image $I_{\theta_{1}\_guide}^{HR}$. We generally select the same channel as the input image or the 0° channel as the guide image.
ii Generate the initial estimate: We select four low-resolution observation images $I_{\theta_{k}}^{LR}$ (*k* = 1, 2, 3, 4) as input images to get initial estimates $T_{\theta_{k}}^{HR}$ through RI or MLRI guide filters.
iii Interpolation in residual domain: We calculate a low-resolution residual image $R_{\theta_{k}}^{LR}$ by making difference between the initial estimate $T_{\theta_{k}}^{HR}$ and the input image $I_{\theta_{k}}^{LR}$. Then, we add the high-resolution residual image $R_{\theta_{k}}^{HR}$ generated by interpolating $R_{\theta_{k}}^{LR}$ and initial estimate $T_{\theta_{k}}^{HR}$ to output the final high-resolution image $I_{\theta_{k}}^{HR}$.

Step (iii) is relatively standardized and fixed in the framework. Thus, we pay major attention to Step (i) and Step (ii). The closer the initial estimate generated in Step (ii) is to the ground truth, the lower the amount of high-frequency information contained in the residual domain, and the better the demosaicing performance of the final high-resolution output image. The initial estimate generated in Step (ii) is the local linear transformation of the guide image generated in Step (i). Therefore, the quality of the guide image directly affects the accuracy of the initial estimation, and further affects the demosaicing performance of the final output image. The quality of the guide image generated in Step (i) is affected by the up-sampling filter and the sampling rate of the observed low-resolution image $I_{\theta_{1}}^{LR}$. We simulated the actual polarization imaging process of DoFP polarimeters using an open-access dataset published in *SPIE Photonics Europe 2018* [55], which includes 10 real-scene 768 × 1024 16-bits near infrared (NIR) images in 0°, 45°, 90°, and 135° polarization directions. We used these simulating images to analyze the influence of the up-sampling filter and sampling rate on the quality of the guide image and the final output image, and further demonstrated the potential of the proposed method to improve the demosaicing performance of the final output image.

### 3.2. Influence of the Up-Sampling Filter

The better the performance of the up-sampling filter, the higher the peak signal to noise ratio (PSNR) of the guide image $I_{\theta_{1}\_guide}^{HR}$ generated in step (i), and the higher the PSNR of the final high-resolution output image. According to the spatial sampling modes of DoFP polarimeters, we down-sampled 10 full-resolution polarization images in 0°, 45°, 90°, and 135° polarization directions of the dataset to generate the observed low-resolution images $I_{0{}^{\circ}}^{LR}$, $I_{45{}^{\circ}}^{LR}$, $I_{90{}^{\circ}}^{LR}$, and $I_{135{}^{\circ}}^{LR}$. Then, we up-sampled $I_{0{}^{\circ}}^{LR}$ by operating step (i) to generate the guide image using 10 up-sampling filters: (a) bilinear interpolation; (b) bicubic interpolation; (c) gradient-based interpolation; (d) Newton polynomial interpolation; (e) up-sampling filter GF1 based on guide filter, where we interpolated $I_{0{}^{\circ}}^{LR}$ using bilinear interpolation to generate the guide image $I_{0{}^{\circ}}^{HR}$, and up-sampled $I_{0{}^{\circ}}^{LR}$ using RI referring to $I_{0{}^{\circ}}^{HR}$ [41]; (f) up-sampling filter GF2 based on guide filter, where we interpolated $I_{0{}^{\circ}}^{LR}$ using bicubic interpolation to generate the guide image $I_{0{}^{\circ}}^{HR}$, and up-sampled $I_{0{}^{\circ}}^{LR}$ using MLRI referring to $I_{0{}^{\circ}}^{HR}$ [43]; (g) up-sampling filter GF3 based on guide filter, where we interpolated $I_{45{}^{\circ}}^{LR}$ using bilinear interpolation to generate the guide image $I_{45{}^{\circ}}^{HR}$, and up-sampled $I_{0{}^{\circ}}^{LR}$ using RI referring to $I_{45{}^{\circ}}^{HR}$; (h) up-sampling filter GF4 based on guide filter, where we interpolated $I_{45{}^{\circ}}^{LR}$ using bicubic interpolation to generate the guide image $I_{45{}^{\circ}}^{HR}$, and up-sampled $I_{0{}^{\circ}}^{LR}$ using MLRI referring to $I_{45{}^{\circ}}^{HR}$; (i) up-sampling filter GF5 based on guide filter, where we interpolated $I_{0{}^{\circ}}^{LR}$ using GF3 to generate the guide image $I_{45{}^{\circ}}^{HR}$, and up-sampled $I_{0{}^{\circ}}^{LR}$ using RI referring to $I_{45{}^{\circ}}^{HR}$, that is, we iterated over (g); (j) up-sampling filter GF6 based on guide filter, where we interpolated $I_{45{}^{\circ}}^{LR}$ using GF4 to generate the guide image $I_{45{}^{\circ}}^{HR}$, and up-sampled $I_{0{}^{\circ}}^{LR}$ using MLRI referring to $I_{45{}^{\circ}}^{HR}$, that is, we iterated over (h).

**Figure 3:**
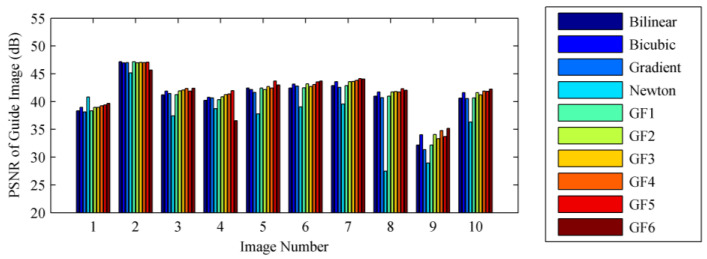
The PSNR of the guide image generated by the 10 up-sampling filters. The abscissa represents the image number in the dataset.

The PSNR of the guide image $I_{0{}^{\circ}\_guide}^{HR}$ generated by the above 10 up-sampling filters is illustrated in Figure 3 Compared with other up-sampling filters, the up-sampling filters GF1–GF6, based on guide filters, perform better in the 10 images of the dataset. We compared GF1–GF6 and found that the performance of the guide filter is extremely dependent on the selected polarization direction of the guide image. The PSNR of $I_{0{}^{\circ}\_guide}^{HR}$, generated by GF1 and GF2, is very close to that generated by bilinear interpolation and bicubic interpolation. That is, the PSNR of the output image generated by GF1 and GF2 is basically the same as the PSNR of the guide image, which means that the guide filter does not show a practical effect. This proves that it is inappropriate to choose the same polarization direction for the guide image and the input image of the guide filter, as in the previously published methods [41,42,43]. We have noticed that when images with different polarization directions are selected as the guide image and the input image (for example, interpolating *I*_0°_ using *I*_45°_ as the guide image in GF3–GF6), the up-sampling filter based on the guide filter can improve the PSNR of $I_{0{}^{\circ}\_guide}^{HR}$.

**Figure 4:**
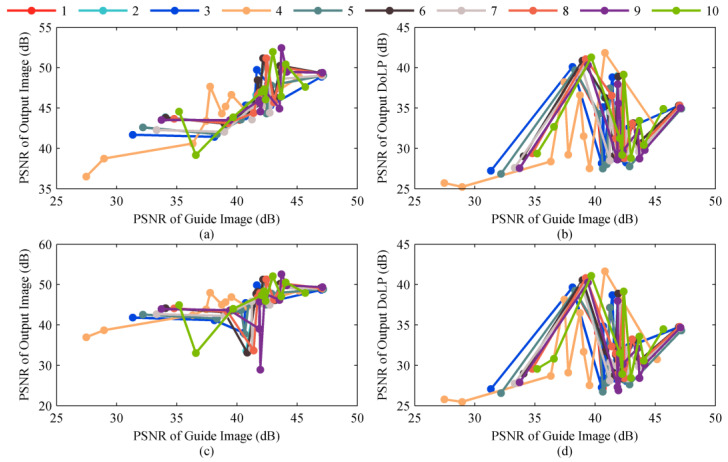
The variation in the PSNR of the high-resolution finial output $I_{45{}^{\circ}}^{HR}$ and the DoLP image with the PSNR of the guide image. The results of 0°, 90°, and 135° are similar to that of 45°, so they are not repeatedly exhibited for conciseness. (**a**,**b**) represents the variation in the PSNR of the high-resolution finial output $I_{45{}^{\circ}}^{HR}$ and the DoLP image calculated by RI with the PSNR of the guide image, respectively. (**c**,**d**) represents the variation in the PSNR of the high-resolution finial output $I_{45{}^{\circ}}^{HR}$ and the DoLP image calculated by MLRI with the PSNR of the guide image, respectively.

Using the guide image $I_{0{}^{\circ}\_guide}^{HR}$ generated by 10 up-sampling filters, we operated steps (ii) and (iii) on $I_{0{}^{\circ}}^{LR}$, $I_{45{}^{\circ}}^{LR}$, $I_{90{}^{\circ}}^{LR}$, and $I_{135{}^{\circ}}^{LR}$ to generate high-resolution output images (we selected the 7 × 7 rectangular window as the filter window) and further calculated the DoLP images (Figure 4). It can be seen that the better the performance of the up-sampling filter, the higher the PSNR of the guide image, and the higher the PSNR of the final high-resolution output image. We compared the results of GF3 and GF5, GF4 and GF6, and found that if we continuously update the guide image through an iterative process and use the output image in the previous iteration as the guide image in the next iteration, the PSNR of the final output image can be increased. Therefore, the proposed method can significantly improve the quality of the output image by selecting an appropriate polarization direction for the guide image and multi-iteration.

### 3.3. Influence of the Sampling Rate of the Guide Image

**Figure 5:**
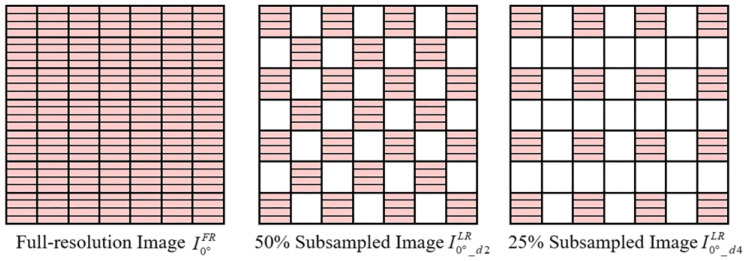
The guide images with different sampling rates.

**Figure 6:**
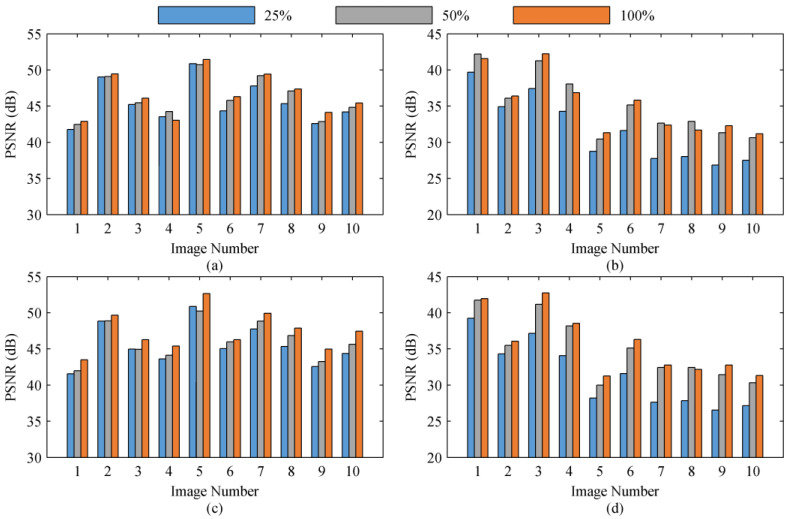
The PSNR of the high-resolution finial output $I_{45{}^{\circ}}^{HR}$ and the DoLP image calculated by three guide images with different sampling rates. The results of 0°, 90°, and 135° are similar to that of 45°, so they are not repeatedly exhibited for conciseness. (**a**,**b**) illustrate the PSNR of $I_{45{}^{\circ}}^{HR}$ and the DoLP calculated by RI, respectively. (**c**,**d**) present the PSNR of $I_{45{}^{\circ}}^{HR}$ and the DoLP calculated by MLRI, respectively.

The higher the sampling rate of the low-resolution observed image $I_{0{}^{\circ}}^{LR}$, the higher the PSNR of the guide image $I_{\theta_{1}\_guide}^{HR}$ generated in Step (i), and the higher the PSNR of the final high-resolution output image. We performed 50% and 25% down-sampling of the full-resolution polarization image $I_{0{}^{\circ}}^{FR}$ in the dataset to generate the low-resolution polarization images $I_{0{}^{\circ}\_d2}^{LR}$ and $I_{0{}^{\circ}\_d4}^{LR}$ (Figure 5). Then, we up-sampled $I_{0{}^{\circ}\_d2}^{LR}$ and $I_{0{}^{\circ}\_d4}^{LR}$ by operating Step (i) to generate $I_{0{}^{\circ}\_d2}^{HR}$ and $I_{0{}^{\circ}\_d4}^{HR}$ using bilinear interpolation. Using $I_{0{}^{\circ}}^{FR}$, $I_{0{}^{\circ}\_d2}^{HR}$ and $I_{0{}^{\circ}\_d4}^{HR}$ as the guide image, we operated Steps (ii) and (iii) on $I_{0{}^{\circ}}^{LR}$, $I_{45{}^{\circ}}^{LR}$, $I_{90{}^{\circ}}^{LR}$, and $I_{135{}^{\circ}}^{LR}$ to generate high resolution output images (we selected the 7 × 7 rectangular window as the filter window) and further calculated the DoLP images (Figure 6).

**Figure 7:**
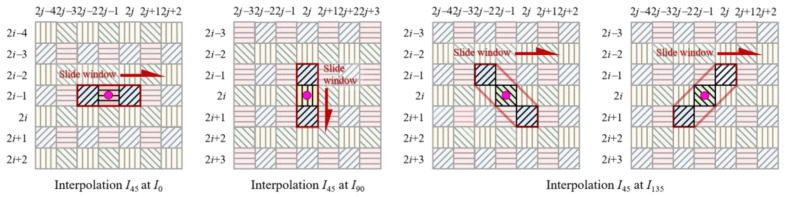
The filter windows with different directions in the guided filter.

It can be seen from Figure 6 that the higher the sampling rate of the low-resolution observed image $I_{\theta_{k}}^{LR}$ used to generate the guide image, the higher the PSNR of the final output high-resolution image. Interpolation for Bayer color camera also follows this rule. When interpolating the R and B channels, we generally choose the interpolation result of the G channel with a higher sampling rate as the guide image. For the DoFP polarimeters, although the 0°, 45°, 90°, and 135° channels have the same sampling rate, the sampling rate of the guide image in the filter window can be increased by choosing the appropriate size and direction for the filtering window in the guide filter. When using the high-resolution $I_{0{}^{\circ}\_guide}^{HR}$ as the guide image to interpolate the missing 45° polarization information at the 0° channel (Figure 7), if we only operate a horizontal guide filter on odd rows and choose a 1 × h rectangular filter window, the sampling rate of the guide image can be increased to 50%. For the same reason, when interpolating the missing 45° polarization information at the 90° channel, we only operate a vertical guide filter on even columns; when interpolating the missing 45° polarization information at the 135° channel, we operate the guide filter along the two diagonal directions, and then fuse the interpolation results in these two directions. According to the spatial layout of the pixeled polarizer array, choosing different channels as the guide image for the pixels in different spatial positions, and cooperating with the different sizes and directions of the filter window, can increase the sampling rate of the guide image in the filter window, and further increase the PSNR of the final output image.

The proposed PAIPRI in this study chooses different channels as the guide image for the pixels in different spatial positions, according to the spatial layout of the pixeled polarizer array. Then, it designs the filter windows with different sizes and directions, and updates the guide image through an iterative process based on the guide filter. The analysis and discussion in this section indicate that the proposed PAIPRI in this study has great potential to improve the demosaicing performance of the final output image compared with the previously published demosaicing method for DoFP polarimeters.

## 4. The Proposed PAIPRI

### 4.1. Overall Pipeline

This study proposed a residual interpolation method, with an integrated pixel-by-pixel adaptive iterative process, for DoFP polarimeters (PAIPRI). Compared with the previously published PRI and MLPRI, the proposed PAIPRI innovatively designed a new guide-image selection strategy, and fused the local iterative optimal results of RI and MLRI. We improved the demosaicing performance of the final output image by increasing the sampling rate and PSNR of the guide image.

**Figure 8:**
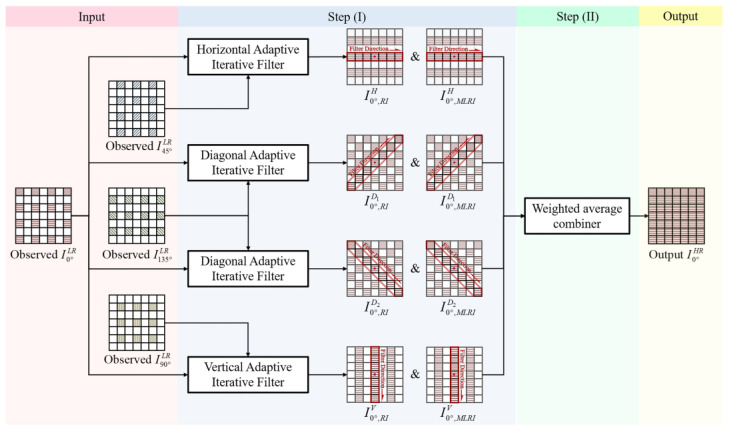
The overall pipeline of the proposed PAIPRI. Horizontal, Vertical, Diagonal Adaptive Iterative Filters represent pixel-by-pixel adaptive iterative processes based on residual interpolation in horizontal, vertical, and two diagonal directions, respectively. Weighted average combiner represents fusion on the interpolation images with RI and MLRI in the horizontal, vertical and two diagonal directions.

The overall pipeline of the proposed PAIPRI is illustrated in Figure 8. We used the up-sampling process of the low-resolution observed image $I_{0{}^{\circ}}^{LR}$ as an example. The up-sampling processes of low-resolution observed images in other polarization directions followed the same principle as that of $I_{0{}^{\circ}}^{LR}$. The proposed PAIPRI consists of two steps:

I Pixel-by-pixel adaptive iterative processes based on residual interpolation in horizontal, vertical, and two diagonal directions: We chose different channels as the guide images for pixels in different spatial positions according to the spatial layout of the pixeled polarizer array, and designed the filter windows with different sizes and directions. When using $I_{0{}^{\circ}}^{LR}$ as the input image, we operated iterative RI and MLRI in horizontal, vertical, and two diagonal directions, referring to the guide images *I*_45°_, *I*_135°_, and *I*_90°_, respectively. In each iterative process, a local criterion was calculated for each reconstructed pixel to adaptively determine whether to update the interpolation result in this iteration. Until all pixels in FPA completed their update or the iterative number reached the maximum iterative number, eight sets of interpolation images, with RI and MLRI in the horizontal, vertical and two diagonal directions, could be obtained.
II According to the spatial layout of the reconstructed pixels, we performed an adaptively weighted average fusion on the eight sets of interpolation images with RI and MLRI in the horizontal, vertical and two diagonal directions to generate the final output up-sampling image, $I_{0{}^{\circ}}^{HR}$.

### 4.2. Pixel-by-Pixel Adaptive Iterative Processes Based on Residual Interpolation

**Figure 9:**
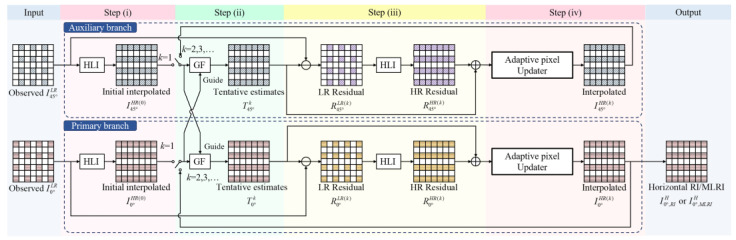
The overall pipeline of the pixel-by-pixel adaptive iterative processes based on residual interpolation in horizontal direction. Primary branch represents the branch up-sampling *I*_0°_, whose final output interpolation results were $I_{0{}^{\circ},RI}^{H}$ and $I_{0{}^{\circ},MLRI}^{H}$. Auxiliary branch, the branch up-sampling the guide image *I*_45°_, whose purpose is updating the guide image of the primary branch and increasing the PSNR of the guide image to further increase the PSNR of $I_{0{}^{\circ},RI}^{H}$ and $I_{0{}^{\circ},MLRI}^{H}$. HLI represents the horizontal linear interpolation. GF represents RI or MLRI. Adaptive pixel updater represents the process of adaptively updating the iterative results, pixel by pixel.

As an example, we used the pixel-by-pixel adaptive iterative processes based on residual interpolation in horizontal direction to interpolate the missing 0° polarization information at the 45° channel (Figure 9). We performed horizontal RI and MLRI, referring to the guide images *I*_0°_ and *I*_45°_. The interpolation result *I*_0°_ and the guide image *I*_45°_ were adaptively updated pixel-by-pixel using two interrelated iterative processes in the primary branch. Then, the high-resolution horizontal interpolation images $RI_{0{}^{\circ}}^{HRH}$ and $MLRI_{0{}^{\circ}}^{HRH}$ were generated. In this step, the guide image, size, and direction of the filter window were selected according to the spatial layout of the pixeled polarizer array of DoFP polarimeters. When interpolating the missing 0° polarization information at the 45° channel, we selected *I*_45°_ as the guide image to operate horizontal RI and MLRI. The sampling rate of the guide image in the filter window was increased to 50% (however, the directionless square window selected in the previously published residual interpolation methods made the sampling rate of the guide image only 25%). The increased sampling rate, cooperating with the iterative process, contributed to the increase in the PSNR of the finial output image. Interpolation of the missing 0° polarization information at the 90° and 135° channels followed the same principle as that at the 0° channel, with the guide image replaced with *I*_90°_ and *I*_135°_ and the filtering direction adjusted to the vertical and two diagonal directions.

The pixel-by-pixel adaptive iterative processes based on residual interpolation consists of four steps:

(i) Calculate the initial value $I_{0{}^{\circ}}^{HR(0)}$ and $I_{45{}^{\circ}}^{HR(0)}$ of the iteration

We performed a horizontal linear interpolation on the observed low-resolution image $I_{0{}^{\circ}}^{LR}$ and $I_{45{}^{\circ}}^{LR}$ to calculate the initial value $I_{0{}^{\circ}}^{HR(0)}$ and $I_{45{}^{\circ}}^{HR(0)}$ of the iteration:

(5) $$\begin{array}{l} I_{0{}^{\circ}}^{HR(0)}\left(2i-1,j\right)=\left\{ \begin{array}{l} \left(I_{0{}^{\circ}}^{LR}\left(2i-1,j-1\right)+I_{0{}^{\circ}}^{LR}\left(2i-1,j+1\right)\right)/2\;\mathrm{if}\;j=2d\;\\ I_{0{}^{\circ}}^{LR}\left(2i-1,j\right)\;\;\;\;\;\;\;\;\;\;\;\;\;\;\;\;\;\;\;\;\;\;\;\;\;\;\;\;\;\;\;\;\;\;\;\;\;\;\;\;\;\;\;\;\mathrm{if}\;j=2d-1 \end{array}\right.,\\ I_{45{}^{\circ}}^{HR(0)}\left(2i-1,j\right)=\left\{ \begin{array}{l} \left(I_{45{}^{\circ}}^{LR}\left(2i-1,j-1\right)+I_{45{}^{\circ}}^{LR}\left(2i-1,j+1\right)\right)/2\;\mathrm{if}\;j=2d-1\\ I_{45{}^{\circ}}^{LR}\left(2i-1,j\right)\;\;\;\;\;\;\;\;\;\;\;\;\;\;\;\;\;\;\;\;\;\;\;\;\;\;\;\;\;\;\;\;\;\;\;\;\;\;\;\;\;\;\;\;\;\mathrm{if}\;j=2d\; \end{array}\right., \end{array}$$

where *d* ∈ *N*^+^; (*i*, *j*) are the pixel indices; *m* ∈ [1, 2*M*], *n* ∈ [1, 2*N*], and 2*M* × 2*N* is the size of the sensor.

In the first iteration, the up-sampling filter was selected as the simple bilinear interpolation. Although the initial iterative value obtained by this simple up-sampling filter was not the best, it greatly simplifies the calculation steps and saves time. The impact of this imperfection in the initial iterative value on the final output images was almost negligible.

(ii) Calculate the initial estimate $T_{0{}^{\circ}}^{k}$ and $T_{45{}^{\circ}}^{k}$

Except for the first iteration, the up-sampling filter in each iteration was the guide filter that was proved to be optimal in Section 3 to increase the PSNR of the final output image. The initial estimate was calculated by the horizontal RI and MLRI through two interrelated iterative processes. In the primary branch, the input and the guide image of the *k*-th iteration, respectively, selected the output of the previous iteration result $I_{0{}^{\circ}}^{HR(k-1)}$ and $I_{45{}^{\circ}}^{HR(k-1)}$ of the primary branch and the auxiliary branch. In the auxiliary branch, the input and the guide image of the *k*-th iteration, respectively, selected the output of the previous iteration result $I_{45{}^{\circ}}^{HR(k-1)}$ and $I_{0{}^{\circ}}^{HR(k-1)}$ of the auxiliary branch and the primary branch. The initial estimate $T_{0{}^{\circ}}^{k}$ and $T_{45{}^{\circ}}^{k}$ by the horizontal RI and MLRI can be expressed as the local linear transformation of the guide image $I_{45{}^{\circ}}^{HR(k-1)}$ and $I_{0{}^{\circ}}^{HR(k-1)}$:

(6) $$\begin{array}{l} T_{0{}^{\circ}}^{k}\left(i,j\right)=a_{0{}^{\circ}}^{k}(m,n)I_{45{}^{\circ}}^{HR(k-1)}\left(i,j\right)+b_{0{}^{\circ}}^{k}(m,n),\forall \left(i,j\right)\in \omega_{mn}^{k},\\ T_{45{}^{\circ}}^{k}\left(i,j\right)=a_{45{}^{\circ}}^{k}(m,n)I_{0{}^{\circ}}^{HR(k-1)}\left(i,j\right)+b_{45{}^{\circ}}^{k}(m,n),\forall \left(i,j\right)\in \omega_{mn}^{k}, \end{array}$$

where $\omega_{mn}^{k}$ represents the filter window selected in the *k*-th iteration; (*m*, *n*) is the index of the center pixel in the filter window $\omega_{mn}^{k}$; *H_k_* × *V_k_* is the size of filter window. In the iterative process, we empirically chose a gradually increasing window size [24]; the window size of the *k*-th iteration was

$$H_{k}=\left\{ \begin{array}{l} \begin{array}{cc} 1 & \mathrm{if}\;k=1\;\mathrm{in}\;\mathrm{RI} \end{array}\\ \begin{array}{cc} 5 & \mathrm{if}\;k=1\;\mathrm{in}\;\mathrm{MLRI} \end{array}\\ \begin{array}{cc} H_{k-1}+2 & \mathrm{if}\;k>1\;\mathrm{in}\;\mathrm{RI}\;\mathrm{and}\;\mathrm{MLRI} \end{array} \end{array}\right.,V_{k}=\left\{ \begin{array}{l} \begin{array}{cc} 5 & \mathrm{if}\;k=1\;\mathrm{in}\;\mathrm{RI}\;\mathrm{and}\;\mathrm{MLRI} \end{array}\\ \begin{array}{cc} V_{k-1}+2 & \mathrm{if}\;k>1\;\mathrm{in}\;\mathrm{RI}\;\mathrm{and}\;\mathrm{MLRI} \end{array} \end{array}\right.,$$

($a_{0{}^{\circ}}^{k}\left(m,n\right)$, $b_{0{}^{\circ}}^{k}\left(m,n\right)$) and ($a_{45{}^{\circ}}^{k}\left(m,n\right)$, $b_{45{}^{\circ}}^{k}\left(m,n\right)$) are linear coefficients, which were assumed to be constant in the filter window with the center pixel (*m*, *n*).

The main difference between RI and MLRI is the different cost functions for solving linear coefficients. When solving linear coefficients in RI, the total difference between the initial estimate $T_{0{}^{\circ}}^{k}$ and $T_{45{}^{\circ}}^{k}$ and the input image $I_{0{}^{\circ}}^{HR(k-1)}$ and $I_{45{}^{\circ}}^{HR(k-1)}$ in the filter window must be minimized. When solving linear coefficients in MLRI, the total Laplacian energy of the difference between the initial estimate $T_{0{}^{\circ}}^{k}$ and $T_{45{}^{\circ}}^{k}$ and the input image $I_{0{}^{\circ}}^{HR(k-1)}$ and $I_{45{}^{\circ}}^{HR(k-1)}$ in the filter window must be minimized to ensure similar image smoothness between the guide image and the initial estimate. RI and MLRI calculate linear coefficients by minimizing the following cost functions in $\omega_{mn}^{k}$, respectively:

(7) $$E\left(a_{0{}^{\circ}}^{k}(m,n),b_{0{}^{\circ}}^{k}(m,n)\right)=\;\left\{ \begin{array}{l} \begin{array}{cc} \sum_{i,j\in \omega_{mn}^{k}}{\left(T_{0{}^{\circ}}^{k}\left(i,j\right)-I_{0{}^{\circ}}^{HR(k-1)}\left(i,j\right)\right)}^{2} & \mathrm{in}\;\mathrm{RI} \end{array}\\ \begin{array}{cc} \sum_{i,j\in \omega_{mn}^{k}}{\left(\Delta \left(T_{0{}^{\circ}}^{k}\left(i,j\right)-I_{0{}^{\circ}}^{HR(k-1)}\left(i,j\right)\right)\right)}^{2} & \mathrm{in}\;\mathrm{MLRI} \end{array} \end{array}\right.,$$

(8) $$E\left(a_{45{}^{\circ}}^{k}(m,n),b_{45{}^{\circ}}^{k}(m,n)\right)=\left\{ \begin{array}{l} \begin{array}{cc} \sum_{i,j\in \omega_{mn}^{k}}{\left(T_{45{}^{\circ}}^{k}\left(i,j\right)-I_{45{}^{\circ}}^{HR(k-1)}\left(i,j\right)\right)}^{2} & \mathrm{in}\;\mathrm{RI} \end{array}\\ \begin{array}{cc} \sum_{i,j\in \omega_{mn}^{k}}{\left(\Delta \left(T_{45{}^{\circ}}^{k}\left(i,j\right)-I_{45{}^{\circ}}^{HR(k-1)}\left(i,j\right)\right)\right)}^{2} & \mathrm{in}\;\mathrm{MLRI} \end{array} \end{array}\right.,$$

where Δ is the operation calculating Laplacian energy, $\Delta I=\left[\begin{array}{ccc} 0 & -1 & 0\\ -1 & 4 & -1\\ 0 & -1 & 0 \end{array}\right]⊗I$, and ⊗ is a convolution operation.

We solved Equations (7) and (8) through linear regression to calculate a set of solutions to linear coefficients:

(9) $$\begin{array}{l} a_{0{}^{\circ}}^{k}\left(m,n\right)=\left\{ \begin{array}{l} \begin{array}{cc} \frac{\frac{1}{C_{mn}^{k}}\sum_{i,j\in \omega_{mn}^{k}}\left(I_{0{}^{\circ}}^{HR(k-1)}\left(i,j\right)\cdot I_{45{}^{\circ}}^{HR(k-1)}\left(i,j\right)\right)-\mu_{0{}^{\circ}}^{k-1}\left(m,n\right)\mu_{45{}^{\circ}}^{k-1}\left(m,n\right)}{{\left(\sigma_{45{}^{\circ}}^{k-1}\left(m,n\right)\right)}^{2}} & \mathrm{in}\;\mathrm{RI} \end{array}\\ \begin{array}{cc} \frac{\sum_{i,j\in \omega_{mn}^{k}}\left(\Delta I_{0{}^{\circ}}^{HR(k-1)}\left(i,j\right)\cdot \Delta I_{45{}^{\circ}}^{HR(k-1)}\left(i,j\right)\right)}{\sum_{i,j\in \omega_{mn}}{\left(\Delta I_{0{}^{\circ}}^{HR(k-1)}\left(i,j\right)\right)}^{2}} & \mathrm{in}\;\mathrm{MLRI} \end{array} \end{array}\right.,\\ \begin{array}{cc} b_{0{}^{\circ}}^{k}\left(m,n\right)=\mu_{0{}^{\circ}}^{k-1}\left(m,n\right)-a_{0{}^{\circ}}^{k}\left(m,n\right)\cdot \mu_{45{}^{\circ}}^{k-1}\left(m,n\right) & \mathrm{in}\;\mathrm{RI}\;\mathrm{and}\;\mathrm{MLRI} \end{array}, \end{array}$$

(10) $$\begin{array}{l} a_{45{}^{\circ}}^{k}(m,n)=\left\{ \begin{array}{l} \begin{array}{cc} \frac{\frac{1}{C_{mn}^{k}}\sum_{i,j\in \omega_{mn}^{k}}\left(I_{0{}^{\circ}}^{HR(k-1)}\left(i,j\right)\cdot I_{45{}^{\circ}}^{HR(k-1)}\left(i,j\right)\right)-\mu_{0{}^{\circ}}^{k-1}(m,n)\mu_{45{}^{\circ}}^{k-1}(m,n)}{{\left(\sigma_{0{}^{\circ}}^{k-1}(m,n)\right)}^{2}} & \mathrm{in}\;\mathrm{RI} \end{array}\\ \begin{array}{cc} \frac{\sum_{i,j\in \omega_{mn}^{k}}\left(\Delta I_{0{}^{\circ}}^{HR(k-1)}\left(i,j\right)\cdot \Delta I_{45{}^{\circ}}^{HR(k-1)}\left(i,j\right)\right)}{\sum_{i,j\in \omega_{mn}}{\left(\Delta I_{45{}^{\circ}}^{HR(k-1)}\left(i,j\right)\right)}^{2}} & \mathrm{in}\;\mathrm{MLRI} \end{array} \end{array}\right.,\\ \begin{array}{cc} b_{45{}^{\circ}}^{k}(m,n)=\mu_{45{}^{\circ}}^{k-1}(m,n)-a_{45{}^{\circ}}^{k}(m,n)\cdot \mu_{0{}^{\circ}}^{k-1}(m,n) & \mathrm{in}\;\mathrm{RI}\;\mathrm{and}\;\mathrm{MLRI} \end{array}, \end{array}$$

where $C_{mn}^{k}$ is the number of whole pixels in $\omega_{mn}^{k}$; $\mu_{0{}^{\circ}}^{k-1}\left(m,n\right)$ and $\sigma_{0{}^{\circ}}^{k-1}\left(m,n\right)$, $\mu_{45{}^{\circ}}^{k-1}\left(m,n\right)$ and $\sigma_{45{}^{\circ}}^{k-1}\left(m,n\right)$ are the mean and standard deviation of $I_{0{}^{\circ}}^{HR(k-1)}$ and $I_{45{}^{\circ}}^{HR(k-1)}$ in $\omega_{mn}^{k}$.

We can calculate a pair of linear coefficients in $\omega_{mn}^{k}$ using Equations (9) and (10). When the filter window traverses all pixels on the FPA, the target pixel is contained in different windows, and corresponds to different linear coefficients. Therefore, we performed a weighted average fusion on these linear coefficients to represent the composite effect of all filter windows containing the target pixel. Then, we calculated the unique pair of linear coefficients corresponding to the target pixel located at (*i*, *j*):

(11) $${\bar{a}}_{0{}^{\circ}}^{k}(i,j)=\frac{\sum_{m,n\in \omega_{ij}^{k}}W_{0{}^{\circ}}(m,n)a_{0{}^{\circ}}^{k}(m,n)}{\sum_{m,n\in \omega_{ij}^{k}}W_{0{}^{\circ}}(m,n)},\;{\bar{b}}_{0{}^{\circ}}^{k}(i,j)=\frac{\sum_{m,n\in \omega_{ij}^{k}}W_{0{}^{\circ}}(m,n)b_{0{}^{\circ}}^{k}(m,n)}{\sum_{m,n\in \omega_{ij}^{k}}W_{0{}^{\circ}}(m,n)},$$

(12) $${\bar{a}}_{45{}^{\circ}}^{k}(i,j)=\frac{\sum_{m,n\in \omega_{ij}^{k}}W_{45{}^{\circ}}(m,n)a_{45{}^{\circ}}^{k}(m,n)}{\sum_{m,n\in \omega_{ij}^{k}}W_{45{}^{\circ}}(m,n)},\;{\bar{b}}_{45{}^{\circ}}^{k}(i,j)=\frac{\sum_{m,n\in \omega_{ij}^{k}}W_{45{}^{\circ}}(m,n)b_{45{}^{\circ}}^{k}(m,n)}{\sum_{m,n\in \omega_{ij}^{k}}W_{45{}^{\circ}}(m,n)},$$

where *W*_0°_ (*m*, *n*) and *W*_45°_ (*m*, *n*) were the corresponding weights of the target pixel in different filter windows.

When calculating linear coefficients (${\bar{a}}_{0{}^{\circ}}^{k}(i,j)$, ${\bar{b}}_{0{}^{\circ}}^{k}(i,j)$) and (${\bar{a}}_{45{}^{\circ}}^{k}(i,j)$, ${\bar{b}}_{45{}^{\circ}}^{k}(i,j)$) using Equations (9)–(12), the output initial estimate of the guide filter can be expressed as:

(13) $$\begin{array}{l} T_{0{}^{\circ}}^{k}\left(i,j\right)={\bar{a}}_{0{}^{\circ}}^{k}(i,j)I_{45{}^{\circ}}^{HR(k-1)}\left(i,j\right)+{\bar{b}}_{0{}^{\circ}}^{k}(i,j),\\ T_{45{}^{\circ}}^{k}\left(i,j\right)={\bar{a}}_{45{}^{\circ}}^{k}(i,j)I_{0{}^{\circ}}^{HR(k-1)}\left(i,j\right)+{\bar{b}}_{45{}^{\circ}}^{k}(i,j), \end{array}$$

(iii) Calculate the residual $R_{0{}^{\circ}}^{HR(k)}$ and $R_{45{}^{\circ}}^{HR(k)}$

The residual represents the difference between the output initial estimates $T_{0{}^{\circ}}^{k}$ and $T_{45{}^{\circ}}^{k}$ of the guide filter and the low-resolution observed image $I_{0{}^{\circ}}^{LR}$ and $I_{45{}^{\circ}}^{LR}$, which can characterize the accuracy of the initial estimates. The low-resolution residual images $R_{0{}^{\circ}}^{LR(k)}$ and $R_{45{}^{\circ}}^{LR(k)}$ can be calculated as:

(14) $$R_{0{}^{\circ}}^{LR(k)}\left(i,j\right)=\left\{ \begin{array}{l} \begin{array}{cc} \left|T_{0{}^{\circ}}^{k}\left(i,j\right)-I_{0{}^{\circ}}^{LR}\left(i,j\right)\right| & \mathrm{if}\;i=2d-1,\;j=2e-1 \end{array}\\ \begin{array}{cc} 0 & otherwise \end{array} \end{array}\right.,$$

(15) $$R_{45{}^{\circ}}^{LR(k)}\left(i,j\right)=\left\{ \begin{array}{l} \begin{array}{cc} \left|T_{45{}^{\circ}}^{k}\left(i,j\right)-I_{45{}^{\circ}}^{LR}\left(i,j\right)\right| & \mathrm{if}\;i=2d-1,\;j=2e \end{array}\\ \begin{array}{cc} 0 & otherwise \end{array} \end{array}\right.,$$

where *d*, *e* ∈ *N*^+^. We calculated high-resolution residual images $R_{0{}^{\circ}}^{HR(k)}$ and $R_{45{}^{\circ}}^{HR(k)}$ by operating a horizontal linear interpolation on low-resolution residual images $R_{0{}^{\circ}}^{LR(k)}$ and $R_{45{}^{\circ}}^{LR(k)}$.

(iv) Pixel-by-pixel adaptively updated iterative results

We defined the following pixel-by-pixel criterion to determine whether to update the interpolation result in the current iteration. For the interpolation result located at (*i*, *j*) of the primary branch and the auxiliary branch in *k*-th iteration, criteria $c_{0{}^{\circ}}^{H(k)}\left(i,j\right)$ and $c_{45{}^{\circ}}^{H(k)}\left(i,j\right)$ were determined:

(16) $$\begin{array}{c} c_{0{}^{\circ}}^{H(k)}(i,j)=a_{0{}^{\circ}}^{H(k)}(i,j)+t_{0{}^{\circ}}^{H(k)}(i,j),\\ a_{0{}^{\circ}}^{H(k)}(i,j)=IF_{Gaussian}\left(\left|T_{0{}^{\circ}}^{k}(i,j)-I_{0{}^{\circ}}^{HR(k-1)}(i,j)\right|\right),\\ t_{0{}^{\circ}}^{H(k)}(i,j)=\frac{1}{C_{mn}^{k}}\sum_{i,j\in \omega_{mn}^{k}}IF_{Gaussian}\left(R_{0{}^{\circ}}^{LR(k)}\left(i,j\right)\right), \end{array}$$

(17) $$\begin{array}{c} c_{45{}^{\circ}}^{H(k)}(i,j)=a_{45{}^{\circ}}^{H(k)}(i,j)+t_{45{}^{\circ}}^{H(k)}(i,j),\\ a_{45{}^{\circ}}^{H(k)}(i,j)=IF_{Gaussian}\left(\left|T_{45{}^{\circ}}^{k}(i,j)-I_{45{}^{\circ}}^{HR(k-1)}(i,j)\right|\right),\\ t_{45{}^{\circ}}^{H(k)}(i,j)=\frac{1}{C_{mn}^{k}}\sum_{i,j\in \omega_{mn}^{k}}IF_{Gaussian}\left(R_{45{}^{\circ}}^{LR(k)}\left(i,j\right)\right), \end{array}$$

where $a_{0{}^{\circ}}^{H(k)}\left(i,j\right)$ and $a_{45{}^{\circ}}^{H(k)}\left(i,j\right)$ describe the convergence of the initial estimates $T_{0{}^{\circ}}^{k}\left(i,j\right)$ and $T_{45{}^{\circ}}^{k}\left(i,j\right)$, obtained from step (ii) in the *k*-th iteration, compared to that obtained in the previous iteration, respectively; $t_{0{}^{\circ}}^{H(k)}\left(i,j\right)$ and $t_{45{}^{\circ}}^{H(k)}\left(i,j\right)$ describe the closeness of the initial estimates $T_{0{}^{\circ}}^{k}\left(i,j\right)$ and $T_{45{}^{\circ}}^{k}\left(i,j\right)$, obtained from step (ii) in the *k*-th iteration, to the observed low-resolution image $I_{0{}^{\circ}}^{LR}$ and $I_{45{}^{\circ}}^{LR}$; *IF_Gaussian_* is the spatial Gaussian filter. We empirically selected a 5 × 5 Gaussian kernel with the standard variation *σ* = 1. The guide filter was a local linear model. Therefore, we used a spatial Gaussian filter to take the influence of neighboring pixels into consideration when calculating the criterion of the target pixel (*i*, *j*), which made the proposed criterion more reliable [24].

Using the criteria calculated from Equation (16), we adaptively updated the iterative results, pixel by pixel, according to the following decision conditions:

(18) $$I_{0{}^{\circ}}^{HR(k)}\left(i,j\right)=\left\{ \begin{array}{l} \begin{array}{cc} T_{0{}^{\circ}}^{k}\left(i,j\right)+R_{0{}^{\circ}}^{HR(k)}\left(i,j\right) & if\;c_{0{}^{\circ}}^{H(k)}(i,j)<\underset{1\leq w\leq k-1}{\min}\left(c_{0{}^{\circ}}^{H(w)}(i,j)\right) \end{array}\\ \begin{array}{cc} I_{0{}^{\circ}}^{HR(k-1)}\left(i,j\right) & otherwise \end{array} \end{array}\right.,$$

(19) $$I_{45{}^{\circ}}^{HR(k)}\left(i,j\right)=\left\{ \begin{array}{l} \begin{array}{cc} T_{45{}^{\circ}}^{k}\left(i,j\right)+R_{45{}^{\circ}}^{HR(k)}\left(i,j\right) & if\;c_{45{}^{\circ}}^{H(k)}(i,j)<\underset{1\leq w\leq k-1}{\min}\left(c_{45{}^{\circ}}^{H(w)}(i,j)\right) \end{array}\\ \begin{array}{cc} I_{45{}^{\circ}}^{HR(k-1)}\left(i,j\right) & otherwise \end{array} \end{array}\right.,$$

where min is the operation calculating minimum value. When the criterion of the *k*-th iteration is smaller than that of the previous *k* − 1 iterations, the interpolation result located at (*i*, *j*) is updated as the sum of the initial estimate $T_{0{}^{\circ}}^{k}\left(i,j\right)$ and $T_{45{}^{\circ}}^{k}\left(i,j\right)$ and the high-resolution residuals $R_{0{}^{\circ}}^{HR(k)}$ and $R_{45{}^{\circ}}^{HR(k)}$. Otherwise, the interpolation result located at (*i*, *j*) is not updated in the *k*-th iteration. When all pixels in FPA complete update, or the iterative number reaches the maximum iterative number *K*, the iterative process stops, and the output $I_{0{}^{\circ},RI}^{H}$ and $I_{0{}^{\circ},MLRI}^{H}$ of step (Ⅰ) are generated.

### 4.3. Fusion on the Iterative Results

According to the spatial layout of the reconstructed pixels, we performed an adaptively weighted average fusion on the eight sets of interpolation images with RI and MLRI in the horizontal, vertical and two diagonal directions obtained in Step (Ⅰ) to generate the finial output up-sampling image $I_{0{}^{\circ}}^{HR}$:

(20) $$I_{0{}^{\circ}}^{HR}(i,j)=\left\{ \begin{array}{l} \begin{array}{cc} I_{0{}^{\circ}}^{LR}(i,j) & if\;i=2d-1,\;j=2e-1 \end{array}\\ \begin{array}{cc} W_{0{}^{\circ},RI}^{H}(i,j)I_{0{}^{\circ},RI}^{H}(i,j)+W_{0{}^{\circ},MLRI}^{H}(i,j)I_{0{}^{\circ},MLRI}^{H}(i,j) & if\;i=2d-1,\;j=2e \end{array}\\ \begin{array}{cc} W_{0{}^{\circ},RI}^{V}(i,j)I_{0{}^{\circ},RI}^{V}(i,j)+W_{0{}^{\circ},MLRI}^{V}(i,j)I_{0{}^{\circ},MLRI}^{V}(i,j) & if\;i=2d,\;j=2e-1 \end{array}\\ \begin{array}{cc} \sum_{w=1}^{2}[W_{0{}^{\circ},MLRI}^{D_{w}}(i,j)I_{0{}^{\circ},RI}^{D_{w}}(i,j)+W_{0{}^{\circ},MLRI}^{D_{w}}(i,j)I_{0{}^{\circ},MLRI}^{D_{w}}(i,j)] & if\;i=2d,\;j=2e \end{array} \end{array}\right.,$$

where *W* is the reciprocal of the minimum value of criteria in 1~*K* iterations. The smaller the criteria, the greater the weight.

$$\begin{array}{l} W_{0{}^{\circ},RI}^{H}(i,j)=1/\underset{1\leq w\leq K}{\min}\left(c_{0{}^{\circ},RI}^{H(w)}(i,j)\right),\;W_{0{}^{\circ},MLRI}^{H}(i,j)=1/\underset{1\leq w\leq K}{\min}\left(c_{0{}^{\circ},MLRI}^{H(w)}(i,j)\right),\\ W_{0{}^{\circ},RI}^{V}(i,j)=1/\underset{1\leq w\leq K}{\min}\left(c_{0{}^{\circ},RI}^{V(w)}(i,j)\right),W_{0{}^{\circ},MLRI}^{V}(i,j)=1/\underset{1\leq w\leq K}{\min}\left(c_{0{}^{\circ},MLRI}^{V(w)}(i,j)\right),\\ W_{0{}^{\circ},RI}^{D_{1}}(i,j)=1/\underset{1\leq w\leq K}{\min}\left(c_{0{}^{\circ},RI}^{D_{1}(w)}(i,j)\right),\;W_{0{}^{\circ},MLRI}^{D_{1}}(i,j)=1/\underset{1\leq w\leq K}{\min}\left(c_{0{}^{\circ},MLRI}^{D_{1}(w)}(i,j)\right),\\ W_{0{}^{\circ},RI}^{D_{2}}(i,j)=1/\underset{1\leq w\leq K}{\min}\left(c_{0{}^{\circ},RI}^{D_{2}(w)}(i,j)\right),\;W_{0{}^{\circ},MLRI}^{D_{2}}(i,j)=1/\underset{1\leq w\leq K}{\min}\left(c_{0{}^{\circ},MLRI}^{D_{2}(w)}(i,j)\right), \end{array}$$

We take the up-sampling process of the observed low-resolution image $I_{0{}^{\circ}}^{LR}$ as an example to illustrate the overall pipeline of the proposed PAIPRI. The up-sampling processes of low-resolution observed images $I_{45{}^{\circ}}^{LR}$, $I_{90{}^{\circ}}^{LR}$, and $I_{135{}^{\circ}}^{LR}$ follow the same principle as that of $I_{0{}^{\circ}}^{LR}$. When up-sampling $I_{45{}^{\circ}}^{LR}$, the guide images in horizontal, vertical and diagonal directions are *I*_0°_, *I*_90°_, and *I*_135°_, respectively. For the same reason, when up-sampling $I_{90{}^{\circ}}^{LR}$, the guide images in horizontal, vertical and diagonal directions are *I*_135°_, *I*_45°_, and *I*_0°_, respectively; when up-sampling $I_{135{}^{\circ}}^{LR}$, the guide images in horizontal, vertical and diagonal directions are *I*_90°_, *I*_0°_, and *I*_45°_, respectively. After completing the up-sampling of the four observed low-resolution images, four high-resolution output images $I_{0{}^{\circ}}^{HR}$, $I_{45{}^{\circ}}^{HR}$, $I_{90{}^{\circ}}^{HR}$, and $I_{135{}^{\circ}}^{HR}$ were generated. Then, we substituted these high-resolution output images into Equations (1)–(4), and reconstructed the high-resolution Stokes vector, DoLP, and AoP images. The whole PAIPRI procedure is presented in (Algorithm 1).
**Alogrithem 1: PAIPRI****Input:** Given the low-resolution observed images of the four polarization direction $I_{0{}^{\circ}}^{LR}$, $I_{45{}^{\circ}}^{LR}$, $I_{90{}^{\circ}}^{LR}$, and $I_{135{}^{\circ}}^{LR}$, the initial value of the window size, and the maximum number of iterations *k_max_*. **Output:** Four high-resolution output images $I_{0{}^{\circ}}^{HR}$, $I_{45{}^{\circ}}^{HR}$, $I_{90{}^{\circ}}^{HR}$, and $I_{135{}^{\circ}}^{HR}$. For *k* = 1:*k_max_*: (i) Calculate the initial iterative value using Equation (5). (ii) Calculate the initial estimate using RI and MLRI in horizontal, vertical, and two diagonal directions for each polarization direction. Solve the linear coefficients using Equations (9)–(12). Then, substitute the linear coefficients and the previous iteration result into Equation (13) to generate the initial estimate in current iteration. (iii) Calculate the residual images in horizontal, vertical, and two diagonal directions for each polarization direction. Substitute the input low-resolution observed image and the initial estimate generated by Step (ii) into Equation (14) to generate the residual images. (iv) Pixel-by-pixel adaptively update iterative results. If criteria in *k*-th iteration < criteria in the previous iteration: Update iterative results in this pixel using Equations (18) and (19). end end (v) Generate the finial output images by adaptively weighting the eight sets of interpolation images with RI and MLRI in the horizontal, vertical and two diagonal directions using Equation (20) after *k* reaches *k_max_* or all the pixels complete updating.

## 5. Experimental Verification and Discussion

This section aims to prove that the proposed PAIPRI exhibits a better demosaicing performance compared with the existing methods for DoFP polarimeters. We compared the demosaicing performance of the proposed PAIPRI with that of the seven existing methods for DoFP polarimeters, including the bilinear interpolation (Bilinear), the bicubic spline interpolation (BS), the gradient-based interpolation (Gradient [35]), the Newton polynomial interpolation (NP [39]), the residual interpolation with minimized residual (PRI [41]), the residual interpolation with minimized Laplacian energy (MLPRI [43]) and edge-aware residual interpolation (EARI [44]). EARI and MLPRI were proven to be the state-of-the-art, non-learning-based polarization demosaicing methods [43,44]. We conducted experiments on both the open-access dataset images [44,55] collected by a division-of-time polarimeter and the indoor and outdoor scene images collected by a real-world DoFP polarimeter demonstrate to analyze and compare the demosaicing performance of the proposed PAIPRI and the seven existing methods in both visual comparison and objective evaluation. It should be noted that the learning-based methods are highly data-dependent. The CNN-based methods in [48,49] did not disclose their training datasets or pre-trained network weights, while the low sample number of open-access datasets [44,55] makes it difficult to produce a satisfactory training result. To ensure fairness, we did not compare these learning-based methods in this section, as in [39,47,48,49,50,51].

### 5.1. Dataset

We used two open-access datasets, published in *SPIE Photonics Europe 2018* [55] and the Morimatsu dataset [44], as the image sources in experiments. According to the spatial sampling modes of DoFP polarimeters, we generated the observed low-resolution images by down-sampling the high-resolution polarization images in 0°, 45°, 90°, and 135° polarization directions of the dataset, which simulated the actual imaging process of DoFP polarimeters. Subsequently, we performed interpolation on these simulated low-resolution observed images using the proposed PAIPRI and six previously published methods. Then, the reconstructed high-resolution polarization images *I*_0°_, *I*_45°_, *I*_90°_, and *I*_135°_ were obtained and substituted into Equations (1)–(4) to reconstruct the high-resolution Stokes vector, DoLP, and AoP images. It should be noted that, when implementing PRI [41] in this section, we used the bilinear interpolation results of the observed low-resolution image instead of the ground-truth image as the guide image [43]. The source codes of EARI [44] were downloaded from the author’s websites, and the error in calculating the Stokes vector was corrected (the pseudo-inverse instead of transpose of M should be used, as shown in Equation (3)).

The polarization demosaicing method aims to obtain an accurate estimate of the unsampled polarization information. It is not sufficient to evaluate a method solely on whether it outputs smooth, visually good results. Therefore, we also carefully analyzed the objective evaluation results. We calculated and compared the PSNR of the reconstructed results for the dataset images. The PSNRs of the reconstructed *I*_0°_, *S*_2_, and DoLP images for dataset [55] are illustrated in Table 1, Table 2 and Table 3 (similarly, there were reconstructed results of *I*_45°_, *I*_90°_, *I*_135°_, *S*_0_, *S*_1_, and AoP, which are not exhibited in order to save space). The highest PSNR of each row in Table 1−3 is shown in bold. The methods using the neighborhood information could not reconstruct the correct information at the boundary of the filled image. Therefore, pixels within 10 pixels from the boundary were excluded in the calculation of PSNR to eliminate the interference of the incorrect information at the boundary in the methods’ performance evaluation. Similar results for the dataset [44] are illustrated in the Supplemental Document (Supplementary Materials). Considering Table 1, Table 2 and Table 3, it can be observed that, for the 10 scene images in the tested dataset, the proposed PAIPRI performs better than the other seven comparison methods in the objective evaluation based on the index PSNR. Compared with the optimal results in the other seven comparison methods, the average PSNR of *I*_0°_, *S*_2_, and DoLP images reconstructed by PAIPRI are increased by 1.33 dB, 1.31 dB, and 0.78 dB, respectively.

We selected three types of representative local regions in the dataset to complete a visual comparison of the demosaicing performance for these eight methods:

(1) Single arc-shaped edge: Due to the difference in material and surface roughness between the target and the background, the boundary in the selected local region 1 appears as continuous and sharp arc-shaped edges in both the intensity images and the polarization images. This type of edge and its neighborhoods in the reconstructed results are easily affected by the IFOV error, and further exhibit a sawtooth effect.
(2) Multi-directional assorted edges: The selected local region 2 contains at least two of the horizontal, vertical, multi-oblique, or arc-shaped edges. This type of edge, and their neighborhoods in the reconstructed results, are easily affected by the IFOV error, and further exhibit sawtooth effect or edge artifacts.
(3) Abundant texture features: The selected local region 3 contains a periodic hole structure. This periodic hole structure appears as a distinct texture feature in both the intensity images and the polarization images. This texture feature is easily affected by the IFOV error in the reconstructed results, and further exhibits additional error textures.

The selected three types of local region can basically cover the image edge and texture features that are susceptible to IFOV errors, and can be used to reasonably evaluate and compare the demosaicing performance for different methods.

**Figure 10:**
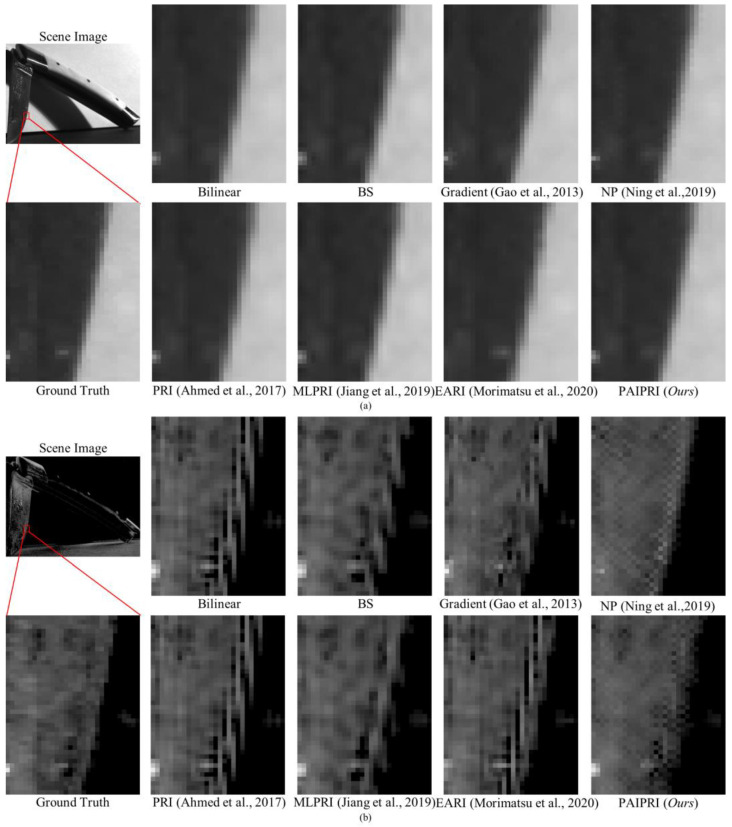
The reconstructed results of the image numbered 4 in the tested dataset, generated by the eight demosaicing methods for DoFP polarimeters [35,39,41,43,44]. The target is a knife composed of a metal body and wooden handle. The background is a rough wall and desktop. To more clearly illustrate the visual differences in the reconstructed results of the seven comparison methods, we zoomed in on a local area marked by a red box with the size of 30 × 40. (**a**) is the *I*_0°_ image. (**b**) is the DoLP image. There are serious sawtooth effects at the single arc-shaped edges reconstructed by Bilinear, BS, Gradient [35], PRI [41] and EARI [44]. The similar results of PRI [41] and bilinear methods, which are also illustrated in Table 1, Table 2 and Table 3, again confirm that it is inappropriate to choose the same polarization direction for the guide image and the input image of the guided filter (as discussed in Section 3.2). NP [39], MLPRI [43] and the proposed PAIPRI can reconstruct sharp edges. However, the reconstructed results of MLPRI [43] appear as excessive smoothing in the target. The reconstructed results of NP [39] generate additional error messages. Although the reconstructed results of PAIPRI retain a small amount of mosaic effect on the edges, it is visually the closest to the ground-truth images.

**Figure 11:**
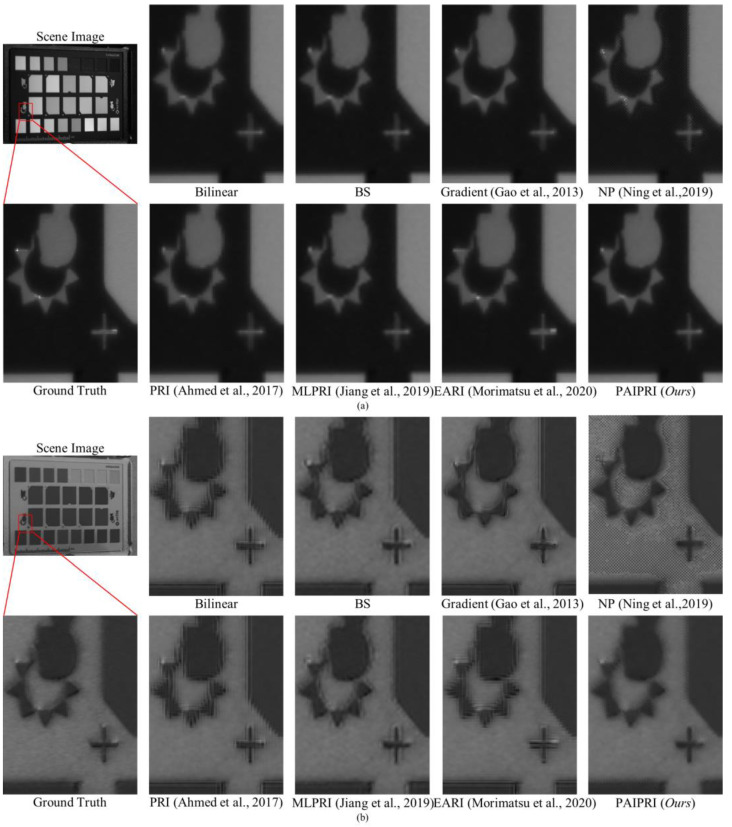
The reconstructed results of the image numbered 8 in the tested dataset generated by the eight demosaicing methods for DoFP polarimeters [35,39,41,43,44]. The target is a standard color checker marked with a white brand logo. We zoomed in on a local area marked by a red box with the size of 90 × 120. (**a**) is the *I*_0°_ image. (**b**) is the DoLP image. There are serious sawtooth effects at the edges reconstructed by Bilinear and PRI [41]. The reconstructed results of BS, Gradient [35], and MLPRI [43] demonstrate blurred edges due to excessive smoothing. The reconstructed results of NP [39] generate a high number of additional error messages in flat-field regions. EARI [44] enhances the horizontal and vertical edges, but the sawtooth effect of edges in other directions is still obvious. However, the proposed PAIPRI can reconstruct clear and sharp edges. Although the reconstructed results of PAIPRI retain a small amount of mosaic effect on horizontal and vertical edges, it is visually the closest to the ground-truth images.

**Figure 12:**
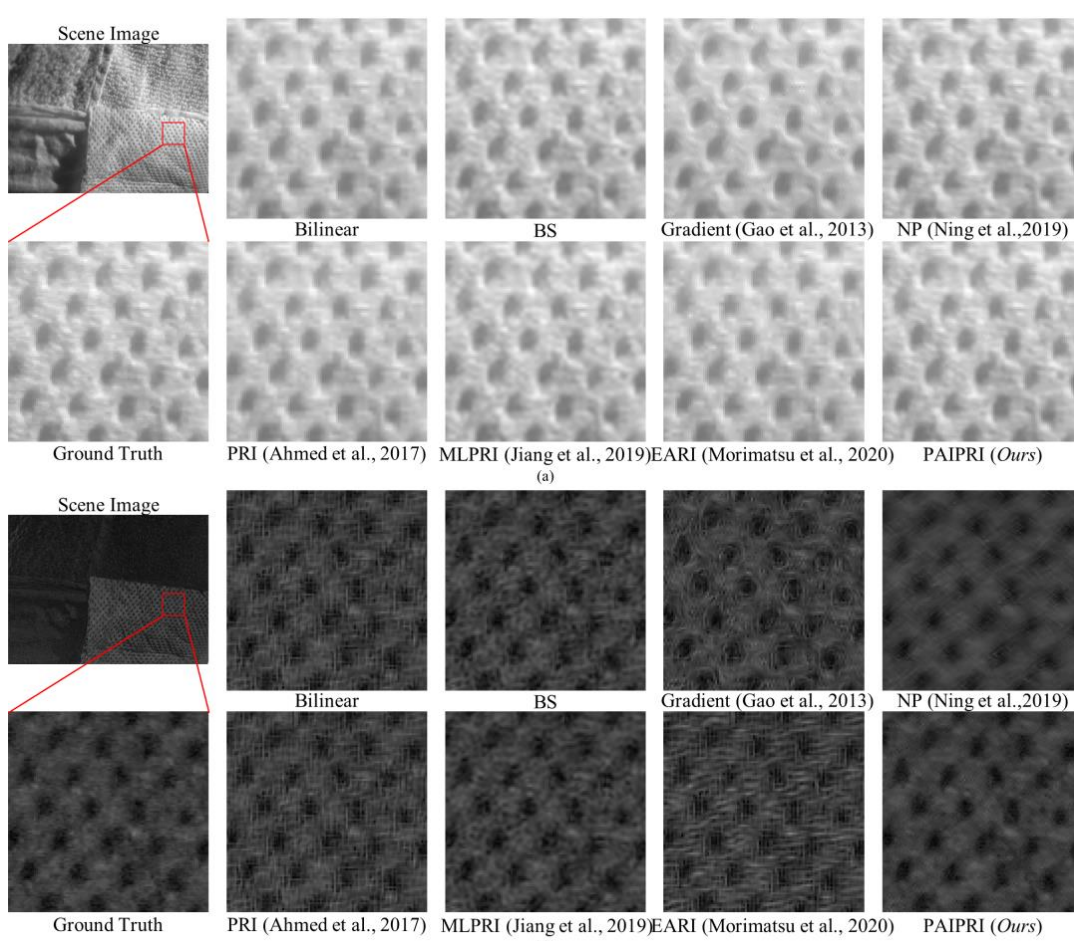
The reconstructed results of the image numbered 1 in the tested dataset generated by the eight demosaicing methods for DoFP polarimeters [35,39,41,43,44]. The target is a fabric with abundant texture features. We zoomed in on a local area marked by a red box with the size of 100 × 100. (**a**) is the *I*_0°_ image. (**b**) is the DoLP image. Bilinear, BS, Gradient [35], PRI [41], MLPRI [43], and EARI [44] cannot reconstruct correct texture features in DoLP images. The reconstructed results of NP [39] demonstrate excessive smoothing. However, the reconstructed results of the proposed PAIPRI can basically reconstruct the correct texture features, and is visually the closest to the ground-truth images.

The reconstructed *I*_0°_, *S*_2_, and DoLP images of the selected three types of representative local regions in the dataset are exhibited in Figure 10, Figure 11 and Figure 12. For the three selected types of representative local regions, the proposed PAIPRI can clearly and accurately reconstruct the edge and texture features, and performed better than the other seven methods in visual comparison, which is consistent with the conclusions obtained from the objective evaluation. For polarization imaging, we apparently paid more attention to the performance of the reconstructed polarization information such as the Stokes vector, the DoLP and the AoP images. However, these images were calculated by the four interpolated polarization channel images, so they are DoLP images, and also support this viewpoint. The proposed PAIPRI still exhibits good demosaicing performance in *S*_2_ and DoLP images (Figure 10a,b, Figure 11a,b and Figure 12a,b). Although the reconstructed results generated by PAIPRI still retains a small amount of mosaic effect on the edges, it did not produce obvious sawtooth effect or edge artifacts at the edges and texture features, nor does it show blurred edges due to excessive smoothing. The reconstructed results generated by PAIPRI are also very visually close to the ground-truth images.

The reconstructed *I*_0°_, *S*_2_, and DoLP images of the selected three types of representative local regions in the dataset are exhibited in Figure 10, Figure 11 and Figure 12. For the selected three types of representative local regions, the proposed PAIPRI can clearly and accurately reconstruct the edge and texture features, and performed better than the other seven methods in terms of visual comparison, which is consistent with the conclusions obtained from the objective evaluation. For polarization imaging, we apparently paid more attention to the performance of the reconstructed polarization information such as the Stokes vector, the DoLP and the AoP images. However, these images were calculated by the four interpolated polarization channel images, so they are DoLP images, and also support this viewpoint. The proposed PAIPRI still exhibited a good demosaicing performance in *S*_2_ and DoLP images (Figure 10a,b, Figure 11a,b and Figure 12a,b). Although the reconstructed results generated by PAIPRI still retain a small amount of mosaic effect on the edges, it did not produce an obvious sawtooth effect or edge artifacts at the edges and texture features, nor did it show blurred edges due to excessive smoothing. The reconstructed results generated by PAIPRI were also very visually close to the ground-truth images.

**Figure 13:**
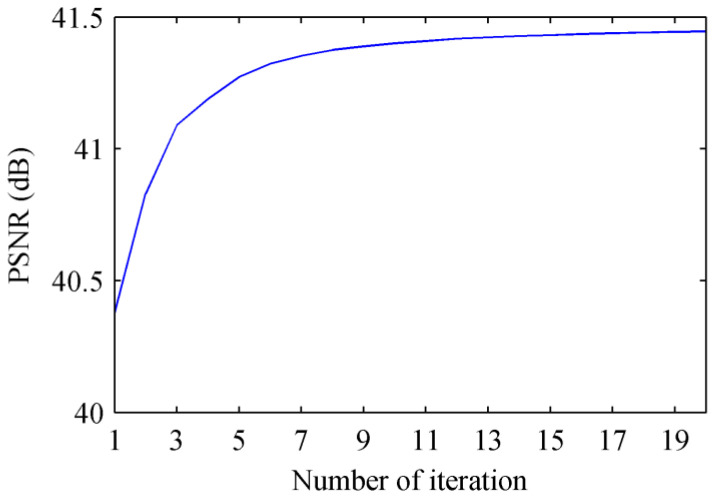
The relationship between the PSNR of *I*_0°_ and the number of iterations.

The computation times of the seven comparison methods and the PAIPRI with different number of iterations are illustrated in Table 4. Figure 13 shows the relationship between the PSNR of *I*_0°_ and the number of iterations. The results shows that, with just five iterations, it is possible to obtain a PSNR that is close to the best one obtained. With more than 15 iterations, the increase in PSNR becomes almost negligible. Compared to other methods, our algorithm has more complex but highly parallelized processing in a single iteration. Thus, the better image quality in a single iteration reduces the number of iterations required for the algorithm to converge. Considering the significant improvement in demosaicing performance, the slight increase in the time needed for the proposed PAIPRI could be a good trade-off.

### 5.2. Images Collected by a Real-World DoFP Polarimeter

**Figure 14:**
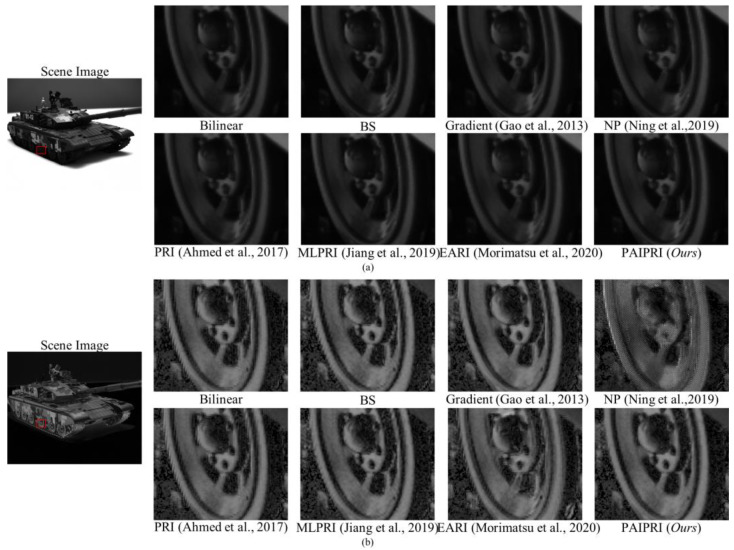
The reconstructed results generated by the eight demosaicing methods on the indoor scene images collected by a real-world DoFP polarimeter [35,39,41,43,44]. The target is a metal tank model with abundant high-frequency information. We zoomed in a local area marked by a red box with the size of 125 × 150. (**a**) is the *I*_0°_ image. (**b**) is the DoLP image. There are serious sawtooth effects at the arc-shaped edges reconstructed by Bilinear, BS, PRI [41], and EARI [44]. The reconstructed results of Gradient [35] demonstrate blurred edges due to excessive smoothing. The reconstructed results of NP [39] generate additional error messages. MLPRI [43] and the proposed PAIPRI can basically reconstruct clear edges, but MLPRI retains some sawtooth effects at the left edge of the wheel. Therefore, PAIPRI is visually the best compared with the other six methods.

**Figure 15:**
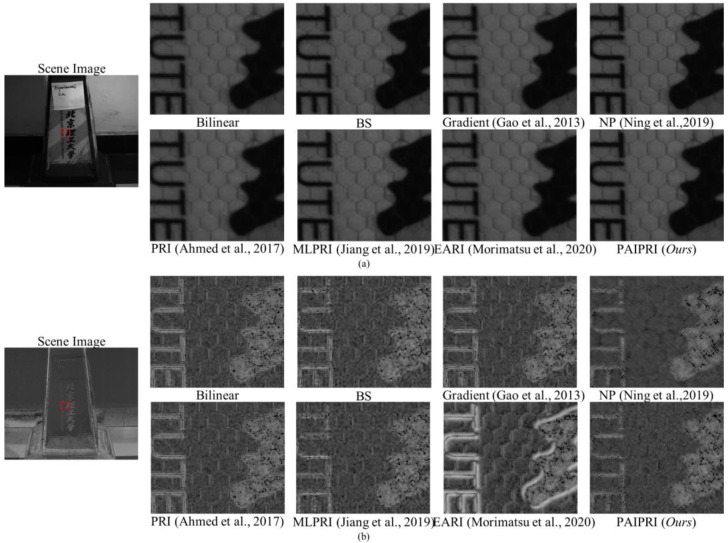
The reconstructed results generated by the eight demosaicing methods on the indoor scene images collected by a real-world DoFP polarimeter [35,39,41,43,44]. The target is a metal tank model with abundant high-frequency information. We zoomed in on a local area marked by a red box with the size of 125 × 150. (**a**) is the *I*_0°_ image. (**b**) is the DoLP image. There are serious sawtooth effects at the arc-shaped edges reconstructed by Bilinear, BS, PRI [41], and EARI [44]. The reconstructed results of Gradient [35] present blurred edges due to excessive smoothing. The reconstructed results of NP [39] generate additional error messages. MLPRI [43] and the proposed PAIPRI can basically reconstruct clear edges, but MLPRI [43] retains some sawtooth effects at the left edge of the wheel. Therefore, PAIPRI is visually the best compared with the other six methods.

We evaluated the demosaicing performance of the proposed PAIPRI through visual comparison using images collected by a real-world DoFP polarimeter. We used the PHX050S DoFP polarimeter of LUCID Vision Labs to collect images, with a sensor size of 2048 × 2448. The adopted image format was 8 bits. The visual comparison of the seven comparison methods using images collected by a real-world DoFP polarimeter are exhibited in Figure 14 and Figure 15. It can be observed that the proposed PAIPRI can clearly and accurately reconstruct the edge and texture features, and performed better than the other six methods in a visual comparison, which is consistent with the conclusions obtained from the objective evaluation and visual comparison of the dataset images.

## 6. Conclusions

Looking at the problems in the selection and preprocessing of the guide image in the previously published residual interpolation methods for DoFP polarimeters, this study proposed a residual interpolation method, with an integrated pixel-by-pixel adaptive iterative process, for DoFP polarimeters. By thoroughly considering the spatial layout of the pixeled polarizer array, we proposed a new guide-image selection strategy, that is, choosing different channels for the pixels in different spatial positions as the guide image, and cooperating with the different sizes and directions of the filter window, which increased the sampling rate of the adopted guide image in the filter window to 50%. Furthermore, the pixel-by-pixel method adaptively updated the guide image and the interpolation result through two interrelated iterative processes and performed an adaptively weighted average fusion on the iterative results of RI and MLRI, which improved the demosaicing performance of the finial output images. Comparison experiments using both the open-access dataset images collected by a DoT polarimeter and the indoor and outdoor scene images collected by a real-world DoFP polarimeter demonstrated that, in a visual comparison, the proposed PAIPRI can reconstruct clear edges and texture features; in an objective evaluation, the average PSNR of *I_0°_*, *S*_2_, and DoLP images reconstructed by PAIPRI are increased by 1.33 dB, 1.31 dB, and 0.78 dB compared with the optimal results in the other seven comparison methods. In brief, the proposed PAIPRI is superior to the existing state-of-the-art methods in terms of both visual comparison and objective evaluation. The results of this study prove that considering the spatial layout of the pixeled polarizer array on the physical level is vital for improving the performances of interpolation methods for DoFP polarimeters.

Redundancy exists between the four polarization channels, which are designed to reduce the noise sensitivity of DoFP polarimeters. Under the condition that the coefficient matrix is known a priori, these four polarization channels satisfy a specific linear relationship. This study has many interesting perspectives, such as using this linear relationship as a reference information source for interpolation and adding more constraints in the process of using the guide filter to solve the initial estimate and the process of residual interpolation. Through these strategies, the interpolation results of the four polarization channels will also meet the linear relationship; additionally, the demosaicing performance is expected to improve.

## Figures and Tables

**Table 1:** PSNR of *I*_0°_ Reconstructed by Different Methods on Dataset.

Image Number	Bilinear	BS	Gradient [35]	NP [39]	PRI [41]	MLPRI [43]	EARI [44]	PAIPRI
1	38.56	39.40	38.39	41.20	38.56	39.40	38.64	**41.40**
2	47.77	47.76	47.77	42.99	47.78	47.76	39.97	**48.21**
3	41.51	42.45	41.93	38.10	41.54	42.50	38.44	**42.97**
4	42.00	42.65	42.44	39.21	42.04	42.71	37.58	**44.42**
5	40.93	40.68	40.16	36.94	40.93	40.68	41.12	**42.14**
6	42.41	43.24	42.80	38.51	42.43	43.31	40.52	**44.81**
7	42.54	43.46	42.27	37.52	42.54	43.46	42.53	**45.00**
8	41.65	42.64	41.46	30.95	41.65	42.64	41.48	**44.44**
9	31.45	33.49	30.62	27.97	31.45	33.52	31.46	**34.38**
10	39.44	40.49	39.34	34.55	39.45	40.52	37.80	**42.05**
Average	40.83	41.63	40.72	36.79	40.84	41.65	38.95	**42.98**

**Table 2:** PSNR of *S_2_* Reconstructed by Different Methods on Dataset.

Image Number	Bilinear	BS	Gradient [35]	NP [39]	PRI [41]	MLPRI [43]	EARI [44]	PAIPRI
1	39.66	42.03	40.63	43.07	39.66	42.53	39.75	**43.87**
2	48.95	49.56	49.19	43.23	48.96	37.66	40.15	**49.59**
3	43.69	45.60	45.32	33.18	43.71	45.43	39.86	**45.87**
4	42.68	44.38	44.08	38.88	42.70	38.80	37.92	**45.78**
5	45.20	45.36	44.80	37.06	45.20	46.03	45.11	**47.30**
6	42.86	44.95	43.93	35.72	42.87	45.23	41.59	**46.49**
7	43.96	47.22	44.16	38.35	43.96	47.69	43.93	**48.25**
8	41.09	44.13	41.24	37.73	41.09	45.12	41.05	**46.12**
9	32.82	35.24	33.05	27.04	32.82	36.41	32.86	**36.78**
10	40.02	43.28	40.86	33.15	40.03	40.11	38.27	**44.86**
Average	42.09	44.18	42.73	36.74	42.10	42.50	40.05	**45.49**

**Table 3:** PSNR of DoLP Reconstructed by Different Methods on Dataset.

Image Number	Bilinear	BS	Gradient [35]	NP [39]	PRI [41]	MLPRI [43]	EARI [44]	PAIPRI
1	41.01	42.67	41.67	42.95	41.01	43.06	40.55	**43.62**
2	37.64	37.70	37.80	28.58	37.65	33.84	35.94	**37.91**
3	39.83	40.96	40.92	34.12	39.83	40.99	38.80	**41.27**
4	37.02	37.90	37.85	36.08	37.03	37.12	35.79	**39.38**
5	31.66	31.49	31.37	29.22	31.66	31.54	30.77	**31.93**
6	34.34	34.66	34.58	23.09	34.35	34.82	33.04	**35.66**
7	31.23	32.52	31.26	20.09	31.23	32.40	30.80	**33.19**
8	31.37	33.03	31.58	18.03	31.37	33.27	31.12	**34.35**
9	28.34	29.77	28.78	16.37	28.34	30.17	28.81	**30.72**
10	30.98	31.56	31.04	25.17	30.98	31.39	30.37	**32.07**
Average	34.34	35.23	34.68	27.37	34.34	34.86	33.60	**36.01**

**Table 4:** Computation Times of Different Methods on Dataset Image Numbered 1.

	Bilinear	BS	Gradient [35]	NP [39]	PRI [41]	MLPRI [43]	EARI [44]	PAIPRI(1)	PAIPRI(3)	PAIPRI(5)	PAIPRI(10)
**Time (s)**	0.07	1.04	1.41	1.37	1.32	2.98	2.52	6.82	22.48	40.84	101.99

## Data Availability

Not applicable.

## Supplementary Materials

The following supporting information can be downloaded at: https://www.mdpi.com/article/10.3390/s22041529/s1, Table S1: PSNR of *I*_0°_ Reconstructed by Different Methods on Dataset.
