# Supplementary material for: Residual Interpolation Integrated Pixel-by-Pixel Adaptive Iterative Process for Division of Focal Plane Polarimeters
Source: Sensors (Basel). 2022 Feb 16;22(4):1529. doi: 10.3390/s22041529 (PMC8874419; doi:10.3390/s22041529)
Supplement: Supplementary file 1 [file sensors-22-01529-s001.zip › sensors-1587274-supplementary.pdf]

Supplemental Document

1. Polarization dataset experimental verification and analysis

We use demosaicing method in DoFP polarization imaging to obtain an accurate estimate of the unsampled polarization information, so as to strictly restore the polarization state of the light wave such as Stokes vector, DoLP, and AoP. Therefore, compared with the visualized comparison focusing on the smooth and visually good visual results, we believe that the objective indicators evaluation deserves more attention. This section aims to prove that the proposed PAIPRI exhibits a better demosaicing performance compared with the existing methods for DoFP polarimeters. We compared the demosaicing performance of the proposed PAIPRI with that of the seven existing methods, including the bilinear interpolation (Bilinear), the bicubic spline interpolation (BS), the gradient-based interpolation (Gradient), the Newton polynomial interpolation (NP), the residual interpolation with minimized residual (PRI), the residual interpolation with minimized Laplacian energy (MLPRI) and edge-aware residual interpolation (EARI). The objective evaluations for Morimatsu dataset images [1] are shown here. Dataset images were collected by a JAI CV-M9GE 3-CCD camera mounted with a SIGMAKOKI SPF-50C-32 linear polarizer. There are 40 real scene 16-bits RGB color images in 0°, 45°, 90°, and 135° polarization directions in this dataset. The size of each polarization image is 768×1024. We selected the raw image in the G channel as the ground truth. According to the spatial sampling modes of the DoFP polarimeters, we down sampled the raw image as the input image of these eight methods. When implementing PRI in this section, we used the bilinear interpolation results of the low-resolution observed image instead of the ground truth image as the guide image [9]. The source codes of EARI [1] are downloaded from the author's websites, and the error in calculating the Stokes vector is corrected (the pseudo-inverse instead of transpose of **M** should be used).

We selected PSNR as the objective evaluation indicator [3–16]. The performance of demosaicing method is affected by the image content. In order to fully evaluate the robustness of these comparison methods to different scene, we displayed the PSNR of each scene image, instead of just giving average PSNR of 40 images. The PSNR of the reconstructed $I_{0°}$, S_2, and DoLP images for dataset [1] are illustrated in Table S1–Table S3 (Similarly, there are reconstructed results of $I_{45°}$, $I_{90°}$, $I_{135°}$, S_0, S_1, and AoP, which are not exhibited to save space). The methods using the neighborhood information cannot reconstruct the correct information at the boundary of the filled image. Therefore, pixels within 10 pixels from the boundary are excluded in calculation of PSNR to eliminate the interference of the incorrect information at the boundary on the methods performance evaluation.

Table S1. PSNR of $I_{0°}$ Reconstructed by Different Methods on Dataset.

Image Number	Bilinear	BS	Gradient [4]	NP [8]	PRI [9]	MLPRI [11]	EARI[1]	PAIPRI
**1**	49.27	50.00	48.57	54.01	49.27	50.00	49.52	**53.59**
**2**	47.22	49.10	46.85	**50.15**	47.22	49.10	45.92	49.05
**3**	38.51	39.27	39.20	41.63	38.52	39.30	37.87	**42.83**
**4**	38.68	39.28	39.03	43.89	38.68	39.29	38.63	**43.05**
**5**	39.09	39.29	38.21	40.63	39.09	39.29	39.20	**40.98**
**6**	31.90	32.63	31.73	34.56	31.90	32.66	31.95	**35.55**
**7**	38.16	39.88	38.93	**41.47**	38.16	39.89	37.84	41.46
**8**	38.98	40.15	39.96	**45.50**	38.99	40.17	38.96	44.51
**9**	43.01	43.68	43.14	**48.18**	43.01	43.68	40.16	48.07
**10**	39.72	40.08	39.64	39.38	39.72	40.08	39.32	**39.97**
**11**	43.06	46.30	43.23	49.37	43.06	46.30	43.57	**49.89**
**12**	43.89	45.39	44.85	46.08	43.89	45.39	43.59	**46.53**
**13**	42.65	45.73	43.35	47.91	42.65	45.77	42.25	**49.07**

**14**	45.79	49.22	47.69	52.68	45.79	49.22	44.97	**52.78**
**15**	36.32	36.26	35.52	**40.73**	36.32	36.28	36.16	39.62
**16**	36.19	36.43	35.29	**40.71**	36.19	36.43	36.30	38.92
**17**	39.01	39.78	39.86	40.45	39.01	39.87	38.82	**42.14**
**18**	44.39	44.72	45.39	**47.64**	44.39	44.73	44.09	45.88
**19**	43.47	44.70	43.69	44.31	43.47	44.70	42.79	**48.44**
**20**	53.63	54.88	55.87	56.45	53.63	54.88	45.43	**57.47**
**21**	41.08	41.98	42.16	44.55	41.08	42.05	41.08	**45.30**
**22**	41.22	41.61	40.97	**44.97**	41.22	41.66	41.01	44.30
**23**	41.78	43.27	42.41	47.07	41.78	43.28	41.33	**47.62**
**24**	39.04	40.63	40.27	**45.94**	39.04	40.63	38.93	45.93
**25**	50.80	51.79	50.02	**55.77**	50.80	51.79	50.46	55.46
**26**	42.36	44.25	43.47	44.16	42.38	44.47	39.55	**45.77**
**27**	41.18	41.94	41.25	44.44	41.18	41.95	41.11	**44.77**
**28**	45.48	47.79	45.89	35.17	45.48	47.79	45.04	**51.16**
**29**	45.63	47.26	45.13	43.11	45.63	47.26	45.33	**50.23**
**30**	36.45	37.53	36.98	**42.53**	36.45	37.53	36.28	41.33
**31**	48.33	50.21	50.23	46.29	48.33	**50.22**	47.10	48.06
**32**	38.92	40.65	38.45	43.54	38.92	40.65	38.83	**44.08**
**33**	38.09	38.56	38.50	**43.81**	38.15	38.62	38.10	42.34
**34**	50.90	52.75	51.91	48.59	50.90	52.75	48.77	**56.55**
**35**	41.10	41.95	40.82	**47.04**	41.10	41.95	40.82	46.74
**36**	41.26	44.01	41.60	43.92	41.28	44.07	40.70	**47.41**
**37**	45.59	46.61	46.18	**51.37**	45.59	46.61	44.61	50.39
**38**	38.36	39.23	37.34	**44.86**	38.36	39.23	38.46	43.87
**39**	47.35	50.25	51.09	54.35	47.35	50.27	45.63	**54.59**
**40**	46.81	49.13	47.46	51.31	46.81	49.13	46.91	**51.76**
**Average**	42.37	43.70	42.80	45.71	42.37	43.72	41.69	**46.69**

Table S2. PSNR of S_2 Reconstructed by Different Methods on Dataset.

Image Number	Bilinear	BS	Gradient [4]	NP [8]	PRI [9]	MLPRI [11]	EARI[1]	PAIPRI
**1**	46.18	46.23	46.31	48.60	46.18	46.83	46.28	**48.52**
**2**	43.91	45.16	43.82	42.47	43.91	**45.37**	42.43	43.50
**3**	34.60	34.82	35.57	33.83	34.60	35.45	34.04	**36.08**
**4**	34.83	34.94	35.39	35.61	34.83	35.45	34.54	**36.11**
**5**	36.48	36.01	36.61	**39.69**	36.48	36.42	36.56	39.07
**6**	29.83	29.95	30.25	29.28	29.83	30.77	29.85	**30.83**
**7**	34.36	35.57	35.31	33.72	34.36	**36.26**	34.08	35.17
**8**	35.93	36.27	37.13	37.42	35.93	36.74	35.96	**37.73**
**9**	39.41	39.65	39.71	41.06	39.41	40.42	36.75	**41.81**
**10**	35.70	35.59	35.95	**38.83**	35.70	35.60	35.13	37.46
**11**	40.11	**42.46**	41.11	41.35	40.11	43.28	40.32	42.11
**12**	40.57	41.41	41.86	41.01	40.57	41.93	40.30	**41.88**
**13**	39.61	42.04	41.15	39.87	39.61	42.71	39.20	**41.65**
**14**	42.72	45.38	44.96	41.78	42.72	**46.16**	42.24	43.74
**15**	32.56	32.06	32.53	**33.96**	32.56	32.86	32.56	33.74
**16**	32.37	32.18	32.14	31.94	32.37	32.50	32.42	**32.74**
**17**	37.33	38.20	38.68	38.84	37.34	39.23	36.95	**40.87**
**18**	42.72	43.03	43.90	44.51	42.72	43.64	41.75	**44.84**
**19**	39.69	40.19	40.43	38.66	39.69	**40.68**	38.99	40.29

**20**	50.75	51.39	53.70	55.11	50.75	52.09	42.78	**54.56**
**21**	37.43	37.61	**38.82**	36.23	37.44	38.23	37.21	38.20
**22**	38.06	37.90	38.55	**39.98**	38.06	38.48	37.92	39.55
**23**	38.01	38.70	39.07	38.43	38.01	39.37	37.70	**39.53**
**24**	36.04	36.77	37.64	37.96	36.04	37.62	35.90	**38.62**
**25**	47.34	47.64	47.35	48.44	47.34	48.32	46.99	**49.04**
**26**	39.05	40.60	40.40	37.46	39.08	**41.42**	36.94	40.15
**27**	38.63	39.16	39.39	37.64	38.63	**39.85**	38.59	39.77
**28**	41.94	43.64	42.73	25.79	41.94	**44.45**	41.43	43.76
**29**	40.95	41.72	40.90	37.50	40.95	**42.45**	40.61	42.21
**30**	32.89	33.44	33.64	34.43	32.89	34.03	32.64	**34.96**
**31**	45.33	46.84	**47.20**	41.85	45.33	46.97	44.09	44.66
**32**	35.13	36.05	35.79	34.25	35.13	**36.48**	35.06	35.63
**33**	34.21	34.31	34.63	35.42	34.26	35.09	34.09	**35.66**
**34**	48.02	49.39	49.14	46.08	48.02	50.30	45.93	**51.14**
**35**	37.58	37.73	37.91	38.86	37.58	38.39	37.30	**39.49**
**36**	38.17	40.16	38.77	38.65	38.17	**40.98**	37.82	40.91
**37**	41.97	42.35	42.65	42.59	41.97	42.87	41.17	**43.35**
**38**	34.75	34.96	34.95	35.92	34.75	35.60	34.78	**36.24**
**39**	44.44	46.56	**48.14**	45.33	44.44	47.50	42.83	47.09
**40**	43.08	44.48	44.05	45.19	43.08	45.05	43.04	**45.65**
**Average**	39.07	39.81	39.96	39.14	39.07	40.45	38.38	**40.71**

Table S3. PSNR of DoLP Reconstructed by Different Methods on Dataset.

Image Number	Bilinear	BS	Gradient [4]	NP [8]	PRI [9]	MLPRI [11]	EARI[1]	PAIPRI
**1**	38.18	38.14	38.54	**41.54**	38.18	39.08	38.48	41.49
**2**	42.48	**43.77**	42.85	41.63	42.48	44.12	38.83	42.25
**3**	30.46	29.93	31.85	28.17	30.46	30.49	29.65	**33.05**
**4**	29.52	28.81	30.37	30.25	29.52	29.14	28.89	**32.01**
**5**	35.41	35.01	35.32	36.98	35.41	35.42	35.64	**37.53**
**6**	29.38	29.06	29.94	28.69	29.38	29.84	29.77	**31.56**
**7**	28.58	26.48	29.45	27.13	28.58	28.15	27.73	**31.65**
**8**	31.40	31.24	33.25	33.09	31.41	31.49	31.26	**34.17**
**9**	31.01	29.92	31.39	31.13	31.01	30.92	26.94	**34.34**
**10**	28.84	27.40	29.28	27.53	28.84	27.63	27.35	**30.29**
**11**	38.18	40.84	39.30	40.29	38.18	**41.80**	38.63	41.21
**12**	31.75	32.57	33.28	31.68	31.75	32.89	31.41	**33.41**
**13**	35.10	37.88	37.06	36.41	35.10	38.38	34.12	**38.50**
**14**	37.58	39.71	40.15	37.81	37.58	**40.43**	35.32	40.12
**15**	29.55	14.31	29.72	29.55	29.55	28.17	29.20	**32.38**
**16**	28.07	27.65	28.28	27.80	28.07	27.96	28.18	**28.83**
**17**	34.91	35.16	36.26	34.03	34.91	36.08	34.53	**37.72**
**18**	36.20	35.59	37.48	34.94	36.20	36.26	35.71	**38.55**
**19**	35.77	36.44	36.97	30.44	35.77	37.06	34.55	**37.57**
**20**	45.01	46.24	49.93	49.27	45.01	46.29	38.33	**50.11**
**21**	35.85	35.13	37.32	34.97	35.85	35.98	34.97	**37.74**
**22**	31.53	30.62	31.92	32.21	31.53	31.14	31.31	**33.03**
**23**	29.26	28.69	30.67	25.40	29.26	29.41	27.88	**32.19**
**24**	29.67	29.66	31.66	32.00	29.67	30.62	28.59	**33.54**
**25**	41.38	41.52	41.70	43.38	41.38	42.17	39.09	**43.51**
**26**	36.69	36.97	38.22	34.90	36.70	38.06	35.50	**38.93**

**27**	29.83	29.70	30.72	31.29	29.83	30.54	29.68	**32.26**
**28**	34.10	35.49	34.83	29.09	34.10	36.49	33.64	**36.97**
**29**	31.12	31.44	31.23	30.97	31.12	32.38	31.06	**33.52**
**30**	26.70	26.20	27.70	28.48	26.70	26.21	26.21	**30.25**
**31**	40.47	41.76	**41.97**	36.13	40.47	42.07	39.02	40.99
**32**	27.75	28.05	28.80	26.99	27.75	28.70	27.13	**29.34**
**33**	29.85	28.76	30.56	30.21	29.85	29.98	29.96	**32.53**
**34**	39.54	41.20	40.78	33.51	39.54	41.77	37.01	**43.79**
**35**	33.41	33.51	34.12	35.66	33.41	34.24	32.94	**36.32**
**36**	34.38	37.79	35.77	32.59	34.38	38.04	31.62	**38.26**
**37**	35.12	35.82	36.56	36.62	35.12	36.19	34.61	**38.20**
**38**	28.39	28.08	28.92	30.55	28.39	28.65	28.41	**31.04**
**39**	34.64	37.15	**40.40**	35.42	34.64	37.83	33.76	38.09
**40**	37.39	38.63	38.50	40.25	37.39	39.47	36.24	**41.20**
**Average**	33.61	33.56	34.83	33.47	33.61	34.54	32.58	**36.21**

It can be observed that for most scene images in the tested dataset, the proposed PAIPRI performs better than the other seven methods in the objective evaluation based on the index PSNR. For images containing large amount of horizontal and vertical edges or textures, the PSNR of polarization channel image $I_{0°}$ reconstructed by NP [8] is slightly higher than that of the proposed PAIPRI method. But the PSNR of S_2, and DoLP images reconstructed by NP [8] is obviously smaller than that of PAIPRI. For polarization imaging, we obviously pay more attention to the performance of the reconstructed polarization information such as the Stokes vector, the DoLP and the AoP images. These reconstructed polarization information images are more susceptible to IFOV errors than the single polarization channels. Compared with the optimal results in the other seven methods, the average PSNR of DoLP images reconstructed by PAIPRI are increased by 1.38dB. This fully demonstrates that considering the layout of the pixeled polarizer array at the physical level plays an important role in IFOV error elimination.

References

1. Morimatsu M.; Monno Y.; Tanaka M.; Okutomi M. Monochrome and Color Polarization Demosaicking Using Edge-Aware Residual Interpolation. In Proceedings of the 27th IEEE International Conference on Image Processing (ICIP), Abu Dhabi, United Arab Emirates, 25–28 October 2020.
2. Lapray P.; Gendre L.; Foulonneau A.; Bigue L. Database of polarimetric and multispectral images in the visible and NIR regions. *Proc. SPIE* **2018**, *10677*, 1067738.
3. Gao S.; Gruev V. Bilinear and bicubic interpolation methods for division of focal plane polarimeters. *Optics Express* **2011**, *19*, 26161–26173.
4. Gao S.; Gruev V. Gradient based interpolation for division of focal plane polarization imaging sensor. In Proceedings of the IEEE International Symposium on Circuits and Systems (ISCAS), Seoul, South Korea, 20–23 May 2012.
5. Zhang J.; Ye W.; Ahmed A.; Qiu Z.; Cao Y.; Zhao X. A Novel Smoothness-Based Interpolation Algorithm for Division of Focal Plane Polarimeters. In Proceedings of the IEEE International Symposium on Circuits & Systems (ISCAS), Baltimore, USA, 28–31 May 2017.
6. Xu X.; Kulkarni M.; Nehorai A.; Gruev V. A correlation-based interpolation algorithm for division-of-focal-plane polarization sensors. *Proc Spie* **2012**, *8364*, 83640L.
7. hang J.; Luo H.; Hui B.; Chang Z. Image interpolation for division of focal plane polarimeters with intensity correlation. *Optics express* **2016**, *24*, 20799–20807.
8. Ning L.; Zhao Y.; Quan P.; Kong S. Demosaicking DoFP images using Newton's polynomial interpolation and polarization difference model. *Optics Express* **2019**, *27*, 1376–1391.
9. Ahmed A.; Zhao X.; Gruev V.; Chang J.; Bermak A. Residual interpolation for division of focal plane polarization image sensors. *Optics Express* **2017**, *25*, 10651–10662.
10. Ahmed A.; Zhao X.; Chang J.; Ma H.; Gruev V.; Bermak A. Four-Directional Adaptive Residual Interpolation Technique for DoFP Polarimeters with Different Micro-polarizer Patterns. *IEEE Sensors Journal* **2018**, *18*, 7990–7997.
11. Jiang T.; Wen D.; Song Z.; Zhang W.; Li Z.; Wei X.; Liu G. Minimized Laplacian residual interpolation for DoFP polarization image demosaicking. *Applied Optics* **2019**, *58*, 7367–7374.
12. Qiu S.; Fu Q.; Wang C.; Heidrich W. Polarization demosaicking for monochrome and color polarization focal plane arrays. In Proceedings of the International Symposium on Vision, Modeling, and Visualization (VMV), Rostock, Germany, 30 September 2019.
13. Zhang J.; Luo H.; Liang R.; Ahmed A.; Zhang X.; Hui B.; Chang Z. Sparse representation-based demosaicing method for microgrid polarimeter imagery. *Optics Letters* **2018**, *43*, 3265–3268.
14. Zhang J.; Shao J.; Luo H.; Zhang X.; Hui B.; Chang Z.; Liang R. Learning a convolutional demosaicing network for microgrid polarimeter imagery. *Optics Letters* **2018**, *43*, 4534–4537.
15. Wen S.; Zheng Y.; Lu F.; Zhao Q. Convolutional demosaicing network for joint chromatic and polarimetric imagery. *Optics Letters* **2019**, *44*, 5646–5649.

16. Zeng X.; Luo Y.; Zhao X.; Ye W. An end-to-end fully-convolutional neural network for division of focal plane sensors to reconstruct S0, DoLP, and AoP. *Optics Express* **2019**, *27*, 8566–8577.
